# An 840 kb distant upstream enhancer is a crucial regulator of catecholamine‐dependent expression of the *Bdnf* gene in astrocytes

**DOI:** 10.1002/glia.24463

**Published:** 2023-08-25

**Authors:** Annela Avarlaid, Eli‐Eelika Esvald, Indrek Koppel, Annabel Parkman, Anna Zhuravskaya, Eugene V. Makeyev, Jürgen Tuvikene, Tõnis Timmusk

**Affiliations:** ^1^ Department of Chemistry and Biotechnology Tallinn University of Technology Tallinn Estonia; ^2^ Protobios LLC Tallinn Estonia; ^3^ Centre for Developmental Neurobiology King's College London London UK

**Keywords:** AP1 transcription factors, astrocyte, BDNF, catecholamines, CREB, enhancer, neurotrophins

## Abstract

Brain‐derived neurotrophic factor (BDNF) plays a fundamental role in the developing and adult nervous system, contributing to neuronal survival, differentiation, and synaptic plasticity. Dysregulation of BDNF synthesis, secretion or signaling has been associated with many neurodevelopmental, neuropsychiatric, and neurodegenerative disorders. Although the transcriptional regulation of the *Bdnf* gene has been extensively studied in neurons, less is known about the regulation and function of BDNF in non‐neuronal cells. The most abundant type of non‐neuronal cells in the brain, astrocytes, express BDNF in response to catecholamines. However, genetic elements responsible for this regulation have not been identified. Here, we investigated four potential *Bdnf* enhancer regions and based on reporter gene assays, CRISPR/Cas9 engineering and CAPTURE‐3C‐sequencing we conclude that a region 840 kb upstream of the *Bdnf* gene regulates catecholamine‐dependent expression of *Bdnf* in rodent astrocytes. We also provide evidence that this regulation is mediated by CREB and AP1 family transcription factors. This is the first report of an enhancer coordinating the transcription of *Bdnf* gene in non‐neuronal cells.

## Main Points

- A ‐840 kb upstream enhancer regulates Bdnf expression in astrocytes.
- The ‐840 kb enhancer is in spatial proximity with Bdnf and facilitates Bdnf expression via CREB and AP1 family transcription factors.

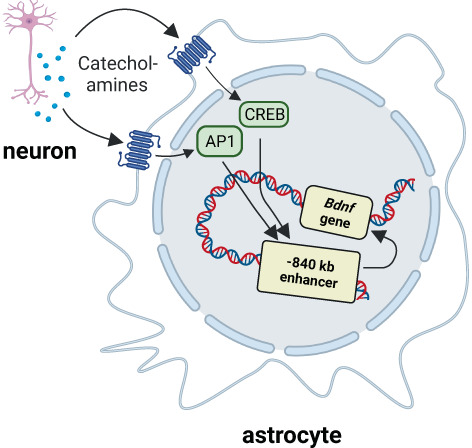

## 1 INTRODUCTION

In recent decades, the study of astrocytes has garnered considerable attention. In addition to their supportive and protective roles, astrocytes contribute to the development and homeostasis of the nervous system (Mederos et al., 2018; Verkhratsky et al., 2016) and modulate synaptic plasticity in the tripartite synapse (Allen, 2014; Farhy‐Tselnicker & Allen, 2018). Similar to neurons, astrocytes respond to a variety of stimuli (Araque & Durkee, 2019), including catecholamine‐dependent signaling in different brain regions in vitro and in vivo (Galloway et al., 2018; Jennings et al., 2017; Pittolo et al., 2022; Vaarmann et al., 2010; Wahis & Holt, 2021). For instance, catecholamine‐evoked signaling in astrocytes modulates synaptic transmission and plasticity (Gordon et al., 2005) to regulate sleep and arousal states (Reitman et al., 2023; Wang et al., 2023), and reward system (Corkrum et al., 2020).

Brain‐derived neurotrophic factor (BDNF) is a member of the neurotrophin family with important functions in the developing and adult nervous system. The expression of the *Bdnf* gene is a result of complex transcriptional regulation: rodent *Bdnf* gene has eight 5′ non‐coding exons (exons I‐VIII) and one 3′ coding exon (exon IX), each of which is controlled by a separate promoter. To produce *Bdnf* transcripts, one of the 5′ non‐coding exons is spliced together with the common 3′ coding exon (Aid et al., 2007; Timmusk et al., 1993). Multiple promoters allow stimulus‐ and tissue‐specific expression of *Bdnf*. Although the regulation of *Bdnf* expression has been studied predominantly in neurons (West et al., 2014), *Bdnf* is also expressed in glial cells (Dai et al., 2003; Elkabes et al., 1996; Koppel et al., 2018; Zafra et al., 1992; Zhang et al., 2014). Astrocytes express *Bdnf* in response to catecholamine signaling (Inoue et al., 1997; Jurič et al., 2006; Koppel et al., 2018; Zafra et al., 1992), indicating that neuron‐astrocyte crosstalk is one of the mechanisms modulating BDNF signaling in the brain. BDNF derived from astrocytes has been shown to promote oligodendrogenesis and provide trophic support after demyelinating lesions (Fulmer et al., 2014; Miyamoto et al., 2015). Moreover, astrocytic BDNF has been shown to modulate the morphology and survival of neurons (De Pins et al., 2019; Giralt et al., 2010, 2011) and is crucial for memory retention (Vignoli et al., 2016).

While the proximal regulatory regions of the *Bdnf* gene have been well described (West et al., 2014), only few studies have investigated the role of different enhancer regions regulating *Bdnf* gene expression in neuronal cells (Beagan et al., 2020; Brookes et al., 2023; Calderon et al., 2022; Flavell et al., 2008; Lyons et al., 2012; Tuvikene et al., 2021). This is a critical knowledge gap considering that enhancer regions refine the spatial, temporal, stimulus and cell type‐specific transcriptional regulation (Nord & West, 2020; Yap & Greenberg, 2018) and alterations in enhancer regions are increasingly associated with diseases (Maurano et al., 2012). Enhancers can be intra‐ or extragenic, located upstream or downstream of a gene, and can be located close or far away from the transcription start sites (TSS) of a gene (Perenthaler et al., 2019). To date, three enhancer regions for *Bdnf* gene have been reported: (1) a MEF2‐binding region located 4.8 kb upstream of *Bdnf* exon I that enhances neuronal activity‐dependent transcription from *Bdnf* promoter I in hippocampal neurons (Flavell et al., 2008) but not in cortical neurons (Lyons et al., 2012); (2) an intronic enhancer region that regulates basal and stimulus‐dependent expression of first cluster of *Bdnf* transcripts in neurons (Tuvikene et al., 2021); and (3) an enhancer located ~237 kb downstream of *Bdnf* exon I that is involved in *Bdnf* expression during neuronal differentiation (Brookes et al., 2023).

We have previously shown that *Bdnf* proximal promoters IV and VI need distal regulatory element(s) for catecholamine‐dependent induction in astrocytes (Koppel et al., 2018). Here, we aimed to identify the regulatory region(s) involved in catecholamine‐dependent *Bdnf* gene transcription in astrocytes. To this end, we first screened several in silico predicted putative *Bdnf* enhancer regions in cortical astrocytes using reporter assays. We characterized a region ~840 kb upstream of *Bdnf* that showed strong dopamine‐dependent bidirectional transcription and potentiated the activity of *Bdnf* promoters in rat cortical astrocytes. We demonstrated that the −840 kb enhancer forms long‐range chromatin interactions with *Bdnf* gene, and further validated its activity in the endogenous context using CRISPR interference and activator systems as well as CRISPR‐mediated deletion of the region. Finally, we show that the activity of the enhancer is regulated by CREB and AP1 family transcription factors. Together, our results provide a novel mechanism for the regulation of *Bdnf* gene in astrocytes.

## 2 MATERIALS AND METHODS

### 2.1 Cultures of rat cortical astrocytes

All animal procedures were performed in accordance with European Directive 2010/63/EU. Animals were maintained under 12 h light/dark cycle in a humidity (50 ± 10%) and temperature (22 ± 1°C) controlled room. Rats were group‐housed (2–4 animals per cage) in conventional polycarbonate or H‐TEMP polysulfone cages with ad libitum access to water and food. Rat cortical astrocyte culture was generated from embryonic (E20/21) Sprague Dawley rats as described previously (Tuvikene et al., 2021).

Briefly, rat cortical astrocytes were grown in 75 cm^2^ cell culture flasks in Dulbecco's Modified Eagle Medium (DMEM) supplemented with 10% fetal bovine serum (Pan Biotech) and 100 U/mL penicillin and 0.1 mg/mL streptomycin (Gibco) at 37°C and 5% CO_2_. At 1 DIV, the whole medium was replaced with fresh supplemented DMEM. At 6 DIV, cell flasks were shaken in a 37°C shaker Certomat® BS‐1 (Sartorius Group) for 18–20 h at 180 rpm. For splitting, medium was removed along with unattached non‐astrocytic cells. Astrocyte monolayer was washed with phosphate‐buffered saline (PBS) and detached from the flask with trypsin–EDTA solution (0.0625% trypsin, 0.25 mM EDTA, Gibco) in 1× PBS at 37°C for 5 min. Astrocytes were collected in supplemented DMEM and centrifuged at 200 *g* for 6 min. Supernatant was aspirated, astrocytes were resuspended in supplemented DMEM, and plated on cell culture plates coated with 0.2 mg/mL poly‐l‐lysine (Sigma‐Aldrich) in Milli‐Q. At 9 DIV, the whole medium was changed. At 15 DIV > 95% of the cells were GFAP‐positive by immunocytochemical analysis and quantification (Figure S1).

### 2.2 Deletion of the −840 kb enhancer region in mouse embryonic stem cells

A2Lox mouse embryonic stem cells (mESCs) were a kind gift from Michael Kyba. Doxycycline‐inducible Neurogenin2 was inserted to A2Lox mESC genome using recombination‐mediated cassette exchange procedure (Iacovino et al., 2011). mESCs were grown and passaged as described in Kainov and Makeyev (2020). The enhancer region located −840 kb from *Bdnf* gene was deleted in the mESCs as described in Tuvikene et al. (2021). Briefly, CRISPR/Cas9 system was used to delete ~500 bp of −840 kb enhancer core region. For that, a total of 6 gRNAs (listed in Supplementary file 1) targeting either upstream or downstream of the mouse −840 kb enhancer core region were designed using the Benchling CRISPR tool, and chosen so that the gRNAs had no predicted off‐targets (maximum of 2 mismatches, allowing both 5′NGG and 5′NAG PAM‐sites) in the protein‐coding regions. The gRNAs were cloned into the pX330 vector (Addgene plasmid #42230). A2Lox‐Neurogenin‐2 mESCs were cotransfected with a mixture of gRNA‐encoding plasmids together with a plasmid containing a blasticidin S deaminase expression cassette. To create control cell lines without any deletion, the mESCs were transfected with pX330 vector containing no gRNA targeting sequence and blasticidin S deaminase‐encoding plasmid. One day after transfection, 8 μg/mL blasticidin S (Sigma‐Aldrich) was added to the media for 3 days to select for transfected cells. The cells were grown for ~2 weeks in 2i media containing LIF and 1% FBS (HyClone™ Fetal Bovine Sera) before selecting single colonies, passaging and genotyping. Deletion of the −840 kb region was determined by PCR and verified using qPCR‐based copy number analysis as described in Tuvikene et al. (2021). PCR amplicons were confirmed with sequencing. All used primers are listed in Supplementary file 1.

### 2.3 Astrocytic differentiation of mouse embryonic stem cells

To produce astrocytes from mESCs, the cells were first differentiated into neuronal progenitor cells (NPCs) based on previously published protocols (Conti et al., 2005; Kleiderman et al., 2016; Pollard et al., 2006; Tiwari et al., 2018) with some modifications. First, 100,000 cells/cm^2^ wildtype mESCs and mESCs with deletion in the −840 kb region were plated on cell culture plates coated with 0.1% gelatin (Sigma‐Aldrich, #ES‐006‐B) and grown for 3 days in N2B27 neuronal differentiation media (DMEM F12‐HAM [Sigma‐Aldrich] and Neurobasal [Invitrogen] 1:1 mixture, 1× in‐house N2, 1× B27 with retinoic acid [Gibco, #17504044], 1× penicillin–streptomycin [Gibco, #15140122], 1 μg/mL laminin [Sigma‐Aldrich, #L2020], 20 μg/mL insulin [Sigma‐Aldrich, #I0516], 500 μM l‐glutamine [Invitrogen]) and media was changed every day. Starting from 4th day, the cells were grown in N2B27/NPC media (DMEM F12‐HAM [Sigma‐Aldrich] and Neurobasal [Invitrogen] 1:1 mixture, 0.5× in‐house N2, 0.5× B27 with retinoic acid [Gibco], 1× penicillin–streptomycin [Gibco, #15140122], 2 mM l‐glutamine [Invitrogen]), and added BSA and insulin as the higher concentrations of BSA (final 37.5 μg/mL) and insulin (final 12.5 μg/mL, Sigma‐Aldrich, #I9278) improve neural cell attachment and survival (Pollard et al., 2006). The used in‐house 100× N2 was prepared as follows: 5 mg/mL BSA (Invitrogen), 2 μg/mL progesterone (Sigma‐Aldrich), 1.6 mg/mL putrescine dihydrochloride (Sigma‐Aldrich), 3 μM sodium selenite (Sigma‐Aldrich), 10 mg/mL apo‐transferrin (Sigma‐Aldrich), 1 mg/mL insulin (Sigma‐Aldrich, #I0516) in DMEM F12‐HAM (Sigma‐Aldrich).

At 7th day, the cells were passaged to produce suspension cultures. For that, the cells were trypsinized with 0.05% trypsin–EDTA (Gibco, #15400054), washed with Neurobasal media, and centrifuged at 300 *g* for 5 min. Finally, the cell pellets were resuspended in NSA media (EuroMed‐N [EuroClone, #ECM0883L], 15 μg/mL insulin [Sigma‐Aldrich, #I9278], and 2 mM l‐glutamine [Invitrogen]) with freshly added 10 ng/mL EGF (Peprotech, #AF‐100‐15) and 10 ng/mL FGF2 (Peprotech, #100‐18B) and plated onto suspension‐culture 6‐well plates (Greiner bio‐one, #657185). Aliquots of growth factors were stored at 4°C and used within 1 week. Fresh media was added to the cells every day and on 3rd day the formed neurospheres were replated to new suspension‐culture 6‐well plates.

On 7th day of suspension culture, the cells were plated to produce adherent monolayer of NPCs. For that, the neurospheres were washed from the well and collected in a tube, centrifuged at 72 *g* for 30 s, resuspended in NSA media with growth factors and plated onto 0.1% gelatin‐coated 6‐well plates. In the next days, the neurospheres sedimented and produced adherent monolayer cultures of NPCs.

Routinely, the cell media was changed every other day and NPCs were passaged when >90% confluency was reached (3rd or 4th day depending on the clonal cell line). For passaging, cell media was collected to a tube, and cells were trypzinised with 0.025% trypsin–EDTA (Gibco, #15400054) at room temperature for 1 min, diluted in 10 times volume of prewarmed DMEM, and collected to the same tube. The cells were centrifuged at 300 *g* for 5 min and resuspended in NSA media with freshly added growth factors. Routinely, 50,000 NPCs/cm^2^ were plated onto 0.1% gelatin‐coated cell culture multi‐well plates.

The identity of the NPCs was verified using RT‐qPCR for *Nes* mRNA (primers are listed in Supplementary file 1) and NESTIN immunocytochemistry using anti‐NES antibody (Developmental Studies Hybridoma Bank, #rat‐401) at 0.2 μg/mL. 96% of the cells were NES‐positive (Figure S6B). To determine *Bdnf* mRNA levels in the NPCs, the clonal cell lines were independently plated at different times and lysed for RNA 2 days after plating.

NPCs were differentiated to astrocytes on PLL‐coated wells (0.2 mg/mL PLL in Milli‐Q) in DMEM with high d‐glucose (Corning) and 1× penicillin–streptomycin (Gibco, #15140122) supplemented with (1) Tet‐system approved 10% FBS only (Gibco, #A4736101); (2) Tet‐system approved 10% FBS (Gibco, #A4736101) with 1× B27 with retinoic acid (Gibco); or (3) Tet‐system approved 10% FBS (Gibco, #A4736101) with 1× B27 with retinoic acid (Gibco) and 1000 U/mL LIF (Sigma‐Aldrich, #ESG1107) for 21 days. The cell media was changed every second day. The identity of the cells was described at different days (1, 5, and 21 days of induction) with RT‐qPCR using primers listed in Supplementary file 1 and by immunocytochemical analysis at 21 days of differentiation. The *Bdnf* mRNA levels were measured with RT‐qPCR using primers listed in Supplementary file 1.

### 2.4 DNA constructs

The selected putative enhancer regions were amplified from genomic DNA and cloned in both forward and reverse orientation in front of the Firefly luciferase reporter gene into pGL4.15 luciferase reporter vector (Promega) without an additional promoter as described previously (Tuvikene et al., 2021). Highly conserved ~500 bp regions from rat, mouse and human −840 kb region were cloned in forward or reverse orientation in front of Firefly luciferase coding sequence in pGL4.15 vector. The coordinates of the cloned regions are listed in Supplementary file 1.

The pRRL‐hPGK‐A‐CREB plasmid has been published previously (Koppel et al., 2018). The coding region of dominant‐negative for AP1 family (A‐FOS) (Ahn et al., 1998), CEBPα (A‐CEBPα), CEBPβ (A‐CEBPβ), USF (A‐USF), and ATF2 (A‐ATF2) (described previously in Esvald et al., 2020) were subcloned to pRRL lentiviral vector under the control of human PGK promoter. The plasmids used to overexpress constitutively active CREB (pQM‐VP16‐CREB) and FOS (pQM‐VP16‐FOS) have been published previously (Pruunsild et al., 2011; Tuvikene et al., 2016). The gRNA targeting sequences (Supplementary file 1) of enhancers were designed using Benchling CRISPR tool (http://www.benchling.com) and cloned into the pRRL‐U6‐gRNA‐hPGK‐EGFP plasmid. We selected gRNAs with high off‐target score (>60) (Hsu et al., 2013) and screened in silico the possible CRISPR gRNA off‐targets based on PAM‐sites (both 5′NGG and 5′NAG) and mismatches (maximum of 2 mismatches), and found no off‐targets for the CRISPR/dCas9 gRNA sequences near the TSS‐s of annotated genes. The lentiviral plasmid pLV‐hUbC‐dCas9‐KRAB‐T2A‐GFP used for CRISPR interference, and pLV‐hUbC‐VP64‐dCas9‐VP64‐T2A‐GFP plasmid used for CRISPR activation have been described previously (Tuvikene et al., 2021). Site‐directed mutagenesis was performed as described previously (Tuvikene et al., 2016) using mutations containing complementary primers (Supplementary file 1).

For CAPTURE‐3C‐sequencing, the FLAG‐tagged biotin acceptor site‐containing nuclease‐deficient Cas9 (FB‐dCas9) and biotin ligase BirA plasmids were obtained from Addgene (plasmid #10054 and #100548) and Liu et al. (2017). The FB‐dCas9 was cloned to pLV backbone under the control of human UbC promoter and BirA was cloned to pRRL backbone under the control of human PGK promoter.

### 2.5 Transfection of cells and luciferase reporter assay

Rat cortical astrocytes were transfected at 13 DIV using 190 ng of luciferase reporter plasmid (or 380 ng of DNA when cotransfected with effector plasmids) and 10 ng of pGL4.83‐SRα‐hRLuc normalizer plasmid (or 20 ng when cotransfected with effector plasmids) in unsupplemented DMEM with a DNA to Lipofectamine 2000 (Invitrogen) ratio of 1:3 (or 1:2 for cotransfection of reporter plasmid and effector plasmid). The transfection was terminated by changing the media to fresh supplemented DMEM.

After treatments at 15 DIV the cortical astrocytes were lysed in 1× Passive Lysis Buffer (Promega) and luciferase assay was performed using Dual‐Glo® Luciferase Assay (Promega) system. Luminescence signal was measured using GENios Pro Multifunction Microplate Reader (Tecan). For data analysis, background corrected Firefly luciferase signals were normalized with background corrected Renilla luciferase signals and the averages of duplicates were calculated.

### 2.6 Lentiviral transduction

The production of lentiviral particles was performed as described previously (Koppel et al., 2018). qPCR was used to determine relative titers of lentiviral particles based on provirus incorporation (primers are listed in Supplementary file 1). For functional experiments, equal amounts of lentiviral particles with >95% transduction efficiency were used to transduce astrocytes. Rat cortical astrocytes were transduced after splitting the astrocytes to 6‐well cell culture plates at 7 DIV.

### 2.7 Drug treatments

Rat cortical astrocytes were treated at 15 DIV and mouse embryonic stem cell‐derived astrocytes were treated on the 21st day of differentiation with 0.15% DMSO as a vehicle control, 150 μM dopamine (Tocris Bioscience) or 25 μM norepinephrine (Tocris Bioscience) in unsupplemented DMEM.

### 2.8 RNA isolation, cDNA synthesis and qPCR

Total RNA from rat cortical astrocytes or mESC‐derived cells was isolated using RNeasy Mini Kit (Qiagen) with on‐column DNase digestion using RNase‐free DNase set (Qiagen) according to the manufacturer's instructions. RNA concentration was measured with BioSpec‐nano spectrophotometer (Shimadzu) or with Nanodrop 2000c spectrophotometer (Thermo Scientific). cDNA was synthesized from equal amounts of total RNA using Superscript III or IV Reverse Transcriptase (Thermo Fisher Scientific) with 1:1 mixture of oligo(dT)_20_ (Microsynth) and random hexamer primers (Microsynth). qPCR was performed in triplicates using 1× HOT FIREpol EvaGreen qPCR Mix Plus (Solis Biodyne) or 1× LightCycler 480 SYBR Green I Master (Roche) on LightCycler 480 II Real Time PCR instrument (Roche). All used qPCR primers are shown in Supplementary file 1. *Ppib* mRNA levels in rat cortical astrocytes and *Cnot4* mRNA levels in mESC‐derived NPCs and astrocytes were used to normalize mRNA and enhancer RNA expression.

### 2.9 Chromatin immunoprecipitation (ChIP)

Rat cortical astrocytes were grown on 10 cm or 15 cm cell culture dishes. After treatments, chromatin was cross‐linked with 1% formaldehyde (methanol‐free, Cell Signaling Technology) for 10 min and quenched with 0.125 M glycine for 10 min at room temperature with gentle agitation. After washing two times with ice‐cold PBS, cells were lysed in 1 mL L1 lysis buffer (50 mM HEPES NaOH [pH 7.5], 140 mM NaCl, 1 mM EDTA, 1 mM EGTA, 0.25% Triton X‐100, 0.5% NP‐40, 10% glycerol, 1× cOmplete protease inhibitor cocktail [Roche]). Cells were collected using cell scraper and centrifuged at 4°C for 5 min at 1400 *g* and the pellets were snap‐frozen and stored at −80°C until further processing. After thawing, the nuclei were washed for 10 min at 4°C in 1 mL L2 buffer (10 mM Tris–HCl [pH 8.0], 200 mM NaCl, 1× cOmplete protease inhibitor cocktail [Roche]), followed by lysis in 500 μL L3 lysis buffer (10 mM Tris–HCl [pH 8.0], 200 mM NaCl, 1 mM EDTA, 0.5 mM EGTA, 0.1% sodium deoxycholate, 0.5% N‐Lauroylsarcosine, 1× cOmplete protease inhibitor cocktail [Roche]) for 5 min. For CREB, pCREB, and FOS ChIP the L1, L2 and L3 lysis buffer also contained phosphatase inhibitors (5 mM NaF, 1 mM beta‐glycerophospatase, 1 mM Na_3_VO_4_, 1 mM Na_4_P_7_O_2_). For histone ChIP, the solutions also contained 10 mM sodium butyrate to inhibit histone deacetylases. Chromatin was sonicated with sonication beads (Diagenode) using 20 cycles (30 s on, 30 s off) on a Bioruptor Pico sonicator (Diagenode) at 4°C. After sonication, lysates were cleared with centrifugation at 4°C for 5 min 10,500 *g*. Protein concentration was measured using BCA Protein Assay Kit (Pierce). Equal amount of protein was taken for ChIP and 10% of the ChIP volume was taken as input samples. The input samples were kept on ice until decrosslinking. 1% of final Triton X‐100 was added to the ChIP samples, and then the samples were diluted 2‐fold in dilution buffer (1% Triton X‐100, 150 mM NaCl, 2 mM EDTA, 20 mM Tris–HCl [pH 8.0], 1× cOmplete protease inhibitor cocktail [Roche]). The ChIP samples were incubated overnight at 4°C with 1 μL (0.031 μg) of H3K27Ac (Cat #8173, Lot #8, Cell Signaling Technology), 1.5 μg of H3K27me3 (Cat #07‐449, Lot 31979, Upstate Biotechnology), 7.5 μL (1.965 μg) of CREB (Cat #07‐449, Lot 4820S, Cell signaling), 7.5 μL (0.435 μg) of pCREB (Cat #87G3, Lot 9198S, Cell signaling) or 1 μL (2 μg) of pan‐FOS (Cat SC‐253x, Lot K0110, Santa Cruz Biotechnology) antibody. At the same time, Dynabeads™ Protein G magnetic beads (Invitrogen) were washed twice with 1 mL PBS‐0.05% Tween‐20 and blocked overnight at 4°C with BSA (200 μg/mL, Thermo Scientific). The following day, beads were washed twice with 1 mL PBS‐0.05% Tween‐20 and diluted in L3 lysis buffer. The beads were added to the ChIP samples and incubated for 5 h while rotating at 4°C. Then, antibody‐bound beads were washed four times with 1 mL cold wash buffer (1% Triton X‐100, 0.1% SDS, 150 mM NaCl, 2 mM EDTA, 20 mM Tris–HCl [pH 8.0], 1× cOmplete protease inhibitor cocktail [Roche]) and once with cold final wash buffer (1% Triton X‐100, 0.1% SDS, 500 mM NaCl, 2 mM EDTA, 20 mM Tris–HCl [pH 8.0], 1× cOmplete protease inhibitor cocktail [Roche]). DNA‐protein complexes were eluted three times with elution buffer (1% SDS, 100 mM NaHCO_3_, 1 mM EDTA) and DNA cross‐links were reversed with 250 mM NaCl at 65°C overnight. The next day, the samples were treated with RNase A (125 μg/mL) for at least 1 h, and then 6 mM EDTA was added and the samples were treated for at least 1 h with Proteinase K (240 μg/mL). The ChIP and input DNA was purified using QIAquick PCR Purification Kit (Qiagen) and DNA enrichment was analyzed with qPCR using 1× LightCycler 480 SYBR Green I Master (Roche). All used qPCR primers are shown in Supplementary file 1. The enrichment of binding was calculated relative to a non‐conserved control region (chr3:99,937,231‐99,937,310, Rn6 genome) located ~5 kbp downstream of the −840 kb enhancer region and selected specifically for this study.

### 2.10 Immunocytochemistry

We used immunocytochemical analyses to characterize NPCs, cultured astrocytes and mESC‐derived induced astrocytes. To analyze induced astrocytes, rat primary mixed culture was used as positive control for antibody staining. These primary cells were obtained from E20‐21 Sprague Dawley rat cortex, plated in DMEM, and grown in supplemented NBA media as described previously (Esvald et al., 2022) without the addition of mitotic inhibitor. NPC‐s, induced astrocytes at 21 days, primary mixed culture at 8 DIV, or cultured astrocytes at 15 DIV were fixed with 4% of paraformaldehyde for 15 min, neutralized and permeabilized with 50 mM NH_4_Cl and 0.5% Triton X‐100 in PBS for another 15 min and blocked with 2% of BSA for at least 30 min. Next, the cells were incubated overnight at 4°C with primary antibody in 0.2% BSA in PBS. The following primary antibodies were used: mouse anti‐GFAP (Sigma‐Aldrich, MAB360, 1:2000), rabbit anti‐GS (Sigma, G2781, 1:2000), rabbit anti‐TUJ1 (Sigma‐Aldrich, T2200, 1:400), mouse anti‐NEUN (Chemicon International, MAB377, 1:100), rabbit anti‐MAP2 (Millipore, AB5622, 1:1000), mouse anti‐NES antibody (Developmental Studies Hybridoma Bank, rat‐401, 1:60), goat anti‐DCX (Santa Cruz, C‐18, 1:500), rabbit anti‐IBA1 (Wako, 019‐19741, 1:1000), and rabbit anti‐CNP (Cell signaling, D83E10, 1:2000). Following the incubation with a primary antibody, cells were washed three times, each time for 5 min, using 1× PBS containing 0.1% Tween‐20.

Goat anti‐mouse Alexa 488 (Invitrogen, 1:2000), goat anti‐rabbit Alexa 594 (Invitrogen, 1:1000), donkey anti‐mouse Alexa 647 (Jackson Immunoresearch, 715‐605‐150, 1:300), and donkey anti‐goat CF488A (Sigma‐Aldrich, 1:2000) were used as secondary antibodies and incubated at room temperature for at least 1 h. Hoechst 33342 (Thermo Scientific, 1:5000) was added to visualize nuclei. After incubation with the secondary antibodies, the cells were washed three times with 1× PBS with 0.1% Tween‐20, and three times with 1× PBS. The cells were imaged using Axiovert 200M (Zeiss) fluorescent microscope (Axio Vision Rel 4.8 program) or LSM 900 (Zeiss) confocal microscope (Zen 3.3 program). The nuclei were counted using ImageJ software and Hoechst 33342 staining. Immunocytochemically labeled cells were counted manually. A total number of at least 300 cells were counted for each antibody staining.

### 2.11 CAPTURE‐3C‐sequencing

At 7 DIV, ~5 × 10^6^ rat cortical astrocytes were transduced with lentiviruses expressing biotin ligase BirA and *Bdnf* promoter VI‐targeting gRNAs 1 and 2 (Supplementary file 1) according to titer measurements and 4‐fold higher dosage of FB‐dCas9‐encoding lentiviruses. The whole media was changed every 2–3 days.

The previously published CAPTURE‐3C‐sequencing protocol (Botten et al., 2023; Liu et al., 2017, 2018, 2020) was adapted for cultured astrocytes. At 15 DIV, after 2 h of treatment, astrocytes grown on 145 mm dishes were washed two times with 1× PBS and chromatin was fixed with 2 mM ethylene glycol‐bis(succinic acid N‐hydroxysuccinimide ester) (EGS, Santa Cruz Biotechnology, #sc‐252807) in PBS with gentle agitation for 45 min. Then 1% formaldehyde (Cell Signaling Technology, #12606S) was added and incubated for additional 10 min. The fixation was quenched with 250 mM glycine for 10 min at room temperature also on an orbital shaker. Next, the astrocytes were washed two times with ice‐cold PBS and scraped in 1 mL ice‐cold cell lysis buffer (25 mM Tris–HCl [pH 7.5], 85 mM KCl, 0.1% Triton X‐100 [Triton™ X‐100 Surfact‐Amps™, Thermo Scientific, #85111], and freshly added 1× cOmplete™ EDTA‐free protease inhibitor cocktail [Roche] and 1 mM DTT [Invitrogen]). The cells were lysed for 30 min while rotating at 4°C. The nuclei were pelleted by centrifugation at 2300 *g* for 8 min at 4°C and the supernatant was removed with a pipette and the pellet was frozen in liquid nitrogen. Freezing the pellet at this stage is recommended as it appears to be an efficient way to help inactivate endonucleases that would otherwise interfere with the protocol.

Each pellet was gently resuspended in 500 μL 1× buffer O (Orange buffer, Thermo Fisher Scientific) and then centrifuged at 2300 *g* for 8 min at 4°C. The pellet was then resuspended in 0.5% SDS and heated at 62°C for 10 min to inactivate endonucleases. Finally, the lysate was cooled for 5 min on ice and SDS was sequestered by adding a final concentration of 1% Triton X‐100 and incubated for 30 min at 37°C. Finally, buffer O (Orange buffer, Thermo Fisher Scientific) and 300 U of DpnII (NEB, #R0543M) were added (as a result, the SDS was also diluted down to 0.1%). Note that the use of buffer O instead of DpnII buffer is critical when performing CAPTURE‐3C‐sequencing from cultured astrocytes, to inhibit the endonucleases present in the cultured cells. DpnII restriction was performed for 4 h while rotating at 37°C. DpnII was inactivated at 65°C for 20 min. Next, the solution was diluted in a Falcon tube 5× up to 3 mL by adding ligase buffer (NEB, final 1×), Triton X‐100 (final concentration 1%), 1× cOmplete™ EDTA‐free protease inhibitor cocktail (Roche), and Milli‐Q. Finally, 2 million CELU units/mL of DNA ligase (NEB, #M0202M) was added per 1 mL. The solution was divided into three DNA LoBind tubes (Eppendorf) and rotated overnight (~16 h) at 16°C.

The next day the ligated chromatin was collected by centrifugation at 3000 *g* for 10 min at 4°C. The pellet was resuspended in 500 μL RIPA 0 buffer (10 mM Tris–HCl [pH 7.5], 1 mM EDTA [pH 8.0], 150 mM NaCl, 0.1% SDS, 1% Triton X‐100, 0.1% sodium deoxycholate, freshly added protease inhibitors, 1 mM DTT), and final concentration of 0.25% N‐lauroylsarcosine. The mixture was sonicated using sonication beads (0.2 g of beads per 0.5 mL solution and prewashed 3× with PBS) for 10 cycles (30 s on and 30 s off) at 4°C with Bioruptor Pico (Diagenode). Finally, the solution was transferred to a new DNA LoBind tube (Eppendorf), centrifuged at 10,500 *g* for 10 min, transferred to another DNA LoBind tube, and final concentration of 300 mM NaCl was added.

For streptavidin pulldown, 100 μL of Pierce™ Streptavidin Magnetic beads (Thermo Scientific, #88817) per one sample was used. The magnetic beads were prewashed two times with 0.1% Tween‐20 in tris‐buffered saline (TBS, pH 7.4). The samples were incubated with streptavidin beads for ~5 h while rotating at 4°C.

Finally, the beads were washed two times with 2% SDS, two times with RIPA buffer with high NaCl (10 mM Tris–HCl [pH 8.0], 1 mM EDTA [pH 8.0], 500 mM NaCl, 0.1% SDS, 1% Triton X‐100, 0.1% sodium deoxycholate), two times with LiCl buffer (250 mM LiCl, 0.5% NP‐40, 0.5% sodium deoxycholate, 10 mM Tris–HCl [pH 8.0], 1 mM EDTA [pH 8.0]), and two times with TE buffer (10 mM Tris–HCl [pH 8.0], 1 mM EDTA [pH 8.0]). DNA was eluted from the beads in proteinase K and SDS elution buffer (1% SDS, 10 mM EDTA [pH 8.0], 50 mM Tris–HCl [pH 8.0], 0.2 mg/mL proteinase K [Thermo Scientific]) overnight at 65°C in a shaker. The next day, DNA was purified with QIAquick PCR Purification Kit (Qiagen). CAPTURE system enrichment to *Bdnf* promoter VI was verified with qPCR using 1× LightCycler 480 SYBR Green I Master (Roche) before library preparation and sequencing. Used qPCR primers are shown in Supplementary file 1.

Library preparation and 150 bp paired‐end sequencing were conducted by Novogene (UK) Company Limited. Sequencing reads (392654811 for DMSO‐treated and 337535553 for DA‐treated cells) were processed and mapped to the Rn6 genome using MAXIM software [accessed 15. June 2023] (Chen et al., 2020). Reads with identical positions in both paired reads were discarded as PCR duplicates. The resultant read pairs were further processed using a model‐based analysis as previously described in Chen et al. (2020) with modifications. For statistical analysis, only data of chromosome 3 was kept. Based on the sequencing read alignment, the region of *Bdnf* promoter VI, coordinates chr3:100,787,200‐100,788,550 on the Rn6 genome, with the FB‐dCas9 peak summit at 100,787,921, was selected as the anchor region. The region ±3 Mb of the anchor region was divided into 20 kb bins with a 5 kb step. For each bin, the number of reads with one end inside the anchor region and the other end in the respective bin was calculated. The distance of each bin from the anchor region was calculated as the distance between the bin midpoint and the FB‐dCas9 peak summit ±10 kb. Read pairs with both ends outside the anchor region were used to define the background interaction distribution for each sample by subsampling *n*_anchor_ read pair distances from chromosome 3 for 10,000 times, where *n*_anchor_ is the number of read pairs with at least one end in the anchor region in the respective sample. Separate negative binomial distributions were fitted for each bin using maximum likelihood estimation, and *p*‐values were calculated from the cumulative distribution function using the count of reads with one end mapped to the anchor region and the other end mapping to the respective bin region. For bins with zero reads interacting with the anchor region, the *p*‐value was set as 1. *p*‐values were corrected for multiple comparisons using Benjamini & Hochberg false discovery rate method. For graphical visualization, the number of anchor‐interacting reads in each bin was divided with the number of *n*_anchor_ reads in the respective sample and multiplied by the mean of *n*_anchor_ reads in both DMSO‐ and DA‐treated samples, to account for slight variations in sequencing depth. Consecutive and overlapping bins with adjusted *p*‐value <.05 were flattened into one interacting region, and maximum read count was used for graphical depiction of the data. All regions analyzed in Capture‐3C‐sequencing are listed in the Supplementary file 1.

### 2.12 Statistical analyses

Sample size estimation was not performed, and randomization and blinding were not used. All tested hypotheses were specified before conducting the experiments. For cultured cells, biological replicates were obtained from rat pups of different litters. For experiments with stem cell‐derived neural cells, independent replicates were individual cell clones. For statistical analysis, normalized data was log transformed, mean centered and autoscaled. Statistical significance was calculated using two‐tailed paired or unpaired *t*‐test, as indicated in figure legends, and *p*‐values were corrected for multiple comparisons with Holm‐Šidak method using Prism 9.5.1 (GraphPad Software). For graphical representation, data was backtransformed and error bars indicate upper and lower limits of backtransformed means ± SEM.

## 3 RESULTS

### 3.1 Selection of putative enhancer regions of *Bdnf* gene in astrocytes

The enhancer regions of *Bdnf* have not been studied in non‐neuronal cells. In addition, previous research from our group suggests that catecholamines induce *Bdnf* expression through a yet undescribed enhancer region(s) in cortical astrocytes (Koppel et al., 2018). In search for the possible enhancers of the *BDNF* gene, we used the GeneHancer subsection of the Genecards database. GeneHancer gathers information about genomic regulatory elements from different human genome‐wide analysis derived from numerous tissues (Fishilevich et al., 2017). Altogether 22 potential regions were predicted as *BDNF* enhancer regions based on the integrated data from several databases (Supplementary file 1). We selected four putative enhancer regions (Table 1, −840, −450, −40, +37 kb) based on the following criteria: (1) high *BDNF* gene‐enhancer score, which indicates the likelihood of a region being an enhancer of the *BDNF* gene; (2) the region is predicted to act as an enhancer in astrocytes; (3) high conservation in mammals; (4) not previously studied as a potential enhancer candidate (Tuvikene et al., 2021); and (5) not located within a promoter region of another gene. All the selected putative enhancer regions were named after their approximate distance from the rat *Bdnf* exon I TSS. The metrics of the GeneHancer database are described in the legend of Table 1.

**TABLE 1:** Potential human *BDNF* enhancer regions predicted by GeneHancer subsection in the GeneCards database and selected for investigation.

Our annotation	GeneHancer identifier	Enhancer score	Enhancer sources	*BDNF* gene‐enhancer score	Total score	TSS distance (kb)	Size (kb)	Transcription factor binding sites within enhancer	Gene targets for enhancer	Coordinates
+37 kb	GH11G027696	1.2	Ensembl	11.1	13.32	+22.7	6.3	92 TFs	3 genes	chr3:100,804,694‐100,805,402 (rn6)
										chr2:109,710,904‐109,711,613 (mm10)
			ENCODE							chr11:27,693,530‐27,694,267 (hg19)
‐40 kb	GH11G027753	0.6	ENCODE	11	6.6	−32.3	1.3	6 TFs	3 genes	chr3:100,727,635‐100,728,249 (rn6)
										chr2:109,626,756‐109,627,366 (mm10)
										chr11:27,775,470‐27,776,121 (hg19)
−840 kb	GH11G028565	1.3	FANTOM5	10.1	13.13	−844.6	2.2	19 TFs	3 genes	chr3:99,932,206‐99,933,054 (rn6)
			Ensembl							chr2:108,827,249‐108,828,106 (mm10)
										chr11:28,587,862‐28,588,875 (hg19)
			ENCODE							
−450 kb	GH11G028145	0.9	Ensembl	5.9	5.31	−424.2	1.2	13 TFs	3 genes	chr3:100,321,518‐100,322,555 (rn6)
										chr2:109,241,562‐109,242,626 (mm10)
			ENCODE							chr11:28,167,349‐28,168,475 (hg19)

*Note*: GeneHancer identifier is a unique tag for the enhancer region in the GeneHancer database. Enhancer score describes the likelihood of the region being an enhancer based on integrated data from different databases, *BDNF* gene‐enhancer score reflects the association between *BDNF* gene expression and the activity of an enhancer, and total score is the multiplication of gene‐enhancer and enhancer scores. Enhancer sources depict datasets that support the region as an enhancer. TSS distance (kb) shows the distance from the human *BDNF* exon I TSS to the midpoint of enhancer, positive values stand for regions downstream of the *BDNF* exon I TSS and negative values show upstream regions. Gene targets for enhancer shows the number of possible target genes. The table also provides the information on the size of the enhancer regions, numbers of transcription factors (TFs) that have been shown to bind the enhancer in ChIP‐seq experiments, and the coordinates of the regions in mouse, rat and human genome are also listed. Our annotation takes into account the distance variations between rat and human genome and is based on the rat genome.

### 3.2 Identification of the region 840 kb upstream of *Bdnf* as a putative dopamine‐regulated *Bdnf* enhancer in cortical astrocytes

**FIGURE 1:**
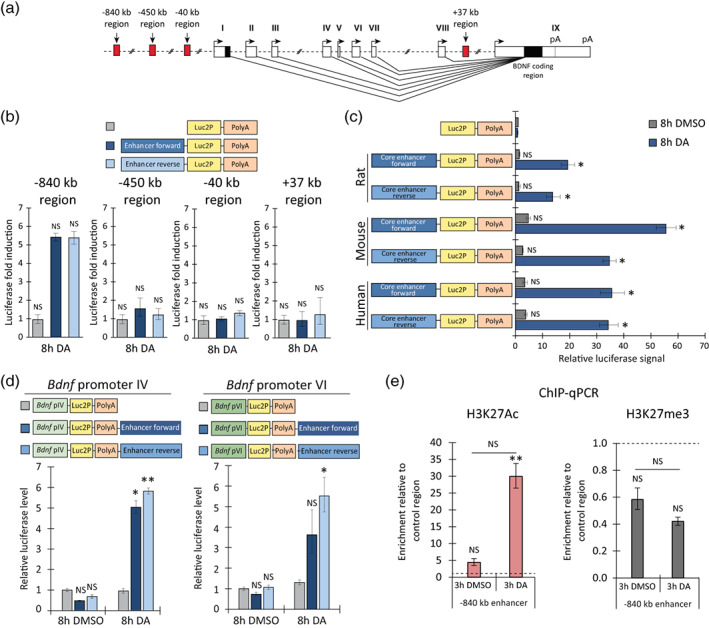
The −840 kb region shows orientation‐independent transcriptional activity and potentiates transcription from *Bdnf* promoters in a dopamine‐dependent manner in cultured rat cortical astrocytes. (a) Schematic representation of rodent *Bdnf* gene structure together with putative enhancer regions screened in luciferase assay. (b–d) Rat cortical astrocytes were transfected at 13 DIV with luciferase reporter vectors indicated on the graphs: rat −840, −450, −40 or +37 kb putative enhancer region in either forward or reverse orientation (b), the conserved core region of either rat, mouse or human −840 kb enhancer in forward or reverse orientation in front of the luciferase coding sequence (Luc2P) followed by polyadenylation signal (PolyA) (c) or luciferase reporter constructs containing rat *Bdnf* promoter IV or VI upstream of the Luc2P coding sequence and the −840 kb region in either forward or reverse orientation downstream of the PolyA (d). Luciferase vector without an enhancer region was used as a negative control. At 15 DIV, transfected astrocytes were treated for 8 h with 0.15% DMSO as a control or 150 μM dopamine (DA). The results are depicted relative to the luciferase signal measured from DMSO‐treated astrocytes transfected with a no enhancer construct (b–d). (e) Histone modifications were analyzed with ChIP‐qPCR assay using anti‐H3K27Ac or anti‐H3K27me3 antibodies. At 15 DIV, astrocytes were treated for 3 h with 0.15% DMSO as a control or 150 μM DA. The region ~5 kb downstream of the −840 kb enhancer was used as a negative control region. Data is depicted as enrichment relative to the control region (dashed line equals to 1). (b–e) Error bars indicate SEM (*n* = 3 independent experiments). Statistical significance was calculated with two‐tailed paired *t*‐test relative to the luciferase level measured from DMSO‐treated cells transfected with luciferase vector without an enhancer (b, c), relative to the luciferase level measured from cells transfected with respective promoter‐containing vector without an enhancer at respective treatment (d), relative to the enrichment of DNA at the negative control region at respective treatment or relative to the respective region in DMSO‐treated astrocytes (e). NS, not significant, *p < .05; **p < .01; ***p < .001, *p*‐values were corrected for multiple comparisons with Holm‐Šidak method.

Kim et al. (2010) described bidirectional transcription of short enhancer‐RNAs (eRNAs) from active enhancer regions and showed that eRNA expression levels are correlated with the expression of target genes, meaning that the transcription from an enhancer region can be used to determine the activity of an enhancer (Kim et al., 2010). First, we used luciferase reporter assay to evaluate whether transcription can start from the putative enhancer regions upon stimuli. We cloned the selected potential rat enhancer regions (−840, −450, −40, +37 kb, Figure 1a,b) as promoters in front of the luciferase reporter gene (without an additional promoter) in either forward or reverse orientation. The cloned enhancer constructs were transfected into rat cortical astrocytes. To see whether these enhancers respond to dopaminergic stimulation, astrocytes were treated with dopamine or DMSO as a vehicle control for 8 h. The −840 kb region showed the highest orientation‐independent induction in response to dopamine treatment (~6‐fold) (Figure 1b). Although the −40 kb region showed the highest bidirectional transcription in basal conditions (Figure S2), the −450, −40 and +37 kb regions did not show dopamine‐dependent induction (Figure 1b), implying that these regions are not dopamine‐dependent enhancer regions in astrocytes.

Functionally important regulatory regions, including enhancers, are known to be evolutionarily conserved (Dickel et al., 2018; Pennacchio et al., 2013). As the −840 kb region showed the strongest transcriptional activation upon stimuli out of the screened candidates, we next inserted the highly conserved ~500 bp long core sequence of the −840 kb region from rat, mouse or human upstream of luciferase coding sequence in either forward or reverse orientation (Figure 1c). In unstimulated astrocytes, mouse and human core enhancer regions upregulated luciferase expression up to ~6‐fold (Figure 1c). Robust upregulation was seen in cortical astrocytes (Figure 1c), where an 8 h dopamine treatment strongly (~14–56‐fold) induced the expression of the reporter gene from all tested −840 kb enhancer core regions. These results demonstrate that the −840 kb region exhibits evolutionarily conserved dopamine‐dependent activity in cortical astrocytes.

Next, we investigated whether the rat −840 kb region could potentiate the activity of *Bdnf* promoters in a heterologous context. For investigation, we chose *Bdnf* promoters IV and VI because *Bdnf* exon IV‐ and exon VI‐containing mRNAs show the highest basal and catecholamine‐induced expression among *Bdnf* transcripts in cortical astrocytes (Koppel et al., 2018). Therefore, we cloned rat *Bdnf* promoters IV or VI in front of the luciferase coding sequence and rat −840 kb enhancer region in forward or reverse orientation downstream of the polyadenylation signal (PolyA) in the same plasmid. Addition of the −840 kb region did not potentiate the activity of *Bdnf* promoter IV or VI (Figure 1d) in unstimulated cells, but strongly increased the stimulus‐dependent activity ~5–6 and ~3–4‐fold, respectively (Figure 1d). Collectively, our results show that the −840 kb region potentiates the dopamine‐dependent activity of *Bdnf* promoters in rat cortical astrocytes.

Finally, to study whether the −840 kb region is an active enhancer region in endogenous context, we interrogated the chromatin state of the region in rat cultured astrocytes. For that, we performed ChIP‐qPCR analysis to investigate the presence of H3K27Ac, a hallmark of active regulatory regions, and H3K27me3, a mark of repressed and condensed chromatin regions (Creyghton et al., 2010; Zhu et al., 2013). Analysis of the −840 kb region showed a ~4‐fold higher enrichment of H3K27Ac relative to control region in unstimulated astrocytes, and a robust ~7‐fold increase in the H3K27Ac mark after 3 h dopamine treatment (Figure 1e). The levels of H3K27me3 were ~2‐fold lower compared to negative control region in both DMSO‐ and dopamine‐treated astrocytes, although this effect failed to reach statistical significance (Figure 1e). To conclude, our results show that the −840 kb region is enriched with an active enhancer chromatin mark in cortical astrocytes especially upon dopamine treatment.

### 3.3 The −840 kb enhancer region is in spatial proximity to *Bdnf* promoter VI in cortical astrocytes

**FIGURE 2:**
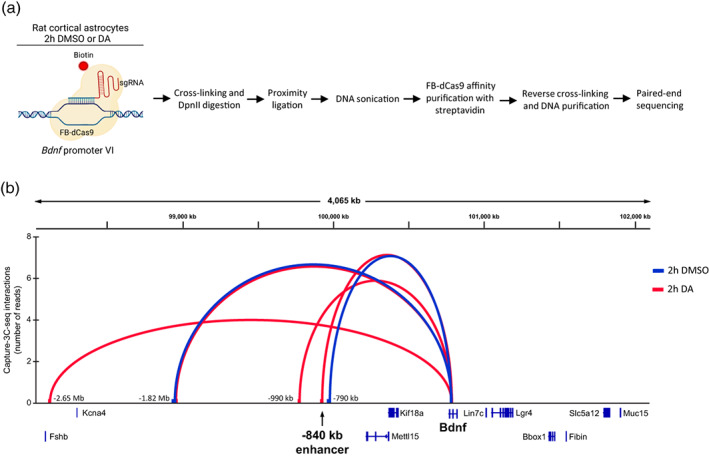
*Bdnf* promoter VI region interacts with various distal genomic regions in cultured cortical astrocytes in a stimulus‐dependent manner. (a) Schematic depiction (created with BioRender.com) of CAPTURE‐3C‐sequencing protocol that was used to determine long‐range chromatin interactions with the *Bdnf* promoter VI region. (b) The statistically significant long‐range interactions of *Bdnf* promoter VI are shown with arcs, and the arc height is proportional to the number of sequencing reads for that interaction. *x*‐axis shows the genomic coordinates in the rat Rn6 genome. Blue—DMSO‐treated cells, red—DA‐treated cells. The −840 kb enhancer region is shown with an arrow. Only interactions with adjusted *p*‐value <.05 are shown. The number of reads and whole statistical analysis of ±3 Mb region of *Bdnf* pVI is shown in Supplementary file 1.

The physical proximity between enhancer and its target promoter has been proposed to be necessary for the activation of transcription (Beagan et al., 2020; Chen et al., 2018). To identify the enhancer regions interacting with the *Bdnf* locus in astrocytes, we used the CAPTURE‐3C‐sequencing method (Botten et al., 2023; Liu et al., 2017, 2018, 2020). Briefly, we used lentivirus‐mediated expression of FLAG‐tagged biotin acceptor site‐containing nuclease deficient Cas9 (FB‐dCas9), biotin ligase BirA, and gRNAs targeting *Bdnf* promoter VI region. We verified that gRNAs targeting *Bdnf* promoter VI did not have a major effect on the *Bdnf* mRNA expression (Figure S3A). The transduced astrocytes were treated with dopamine or DMSO as a vehicle control for 2 h and analyzed by CAPTURE‐3C‐sequencing (Figure 2a). The enrichment of *Bdnf* promoter VI was verified using qPCR before next‐generation sequencing (Figure S3B). Our CAPTURE‐3C‐sequencing results also showed a strong enrichment of the *Bdnf* pVI region (Figure S3C). In total, we determined 10,559 and 9066 sequencing reads with at least one end in the *Bdnf* pVI anchor region, for DMSO‐ and DA‐treated cells, respectively and of these, 220 and 249 reads, respectively showed long‐range intrachromosomal interaction outside the pVI anchor region.

The CAPTURE‐3C‐sequencing experiment revealed that the *Bdnf* promoter VI region exhibits a few statistically significant interactions with distant upstream regions. Specifically, we identified a stimulus‐independent interaction with a region located 1.82 Mb upstream of *Bdnf* gene. Following dopamine treatment, we observed alterations in the three‐dimensional structure of chromatin. Namely, an interaction with the −790 kb region was exclusively detected in DMSO‐treated cells, while DA‐induced interactions were noted with the −840 kb, −990 kb and −2.65 Mb regions. We did not detect any interactions near the *Bdnf* gene locus, even when looking with a smaller bin size of 5 kb (data not shown). Interestingly, we did not find any interactions downstream of the *Bdnf* gene in astrocytes. Collectively, our findings demonstrate that the −840 kb region physically associates with the *Bdnf* promoter VI in astrocytes in a catecholamine signaling‐dependent manner, supporting the role of the −840 kb region as a stimulus‐dependent enhancer of the *Bdnf* gene.

### 3.4 The −840 kb enhancer regulates catecholamine‐induced *Bdnf* expression in cortical astrocytes

**FIGURE 3:**
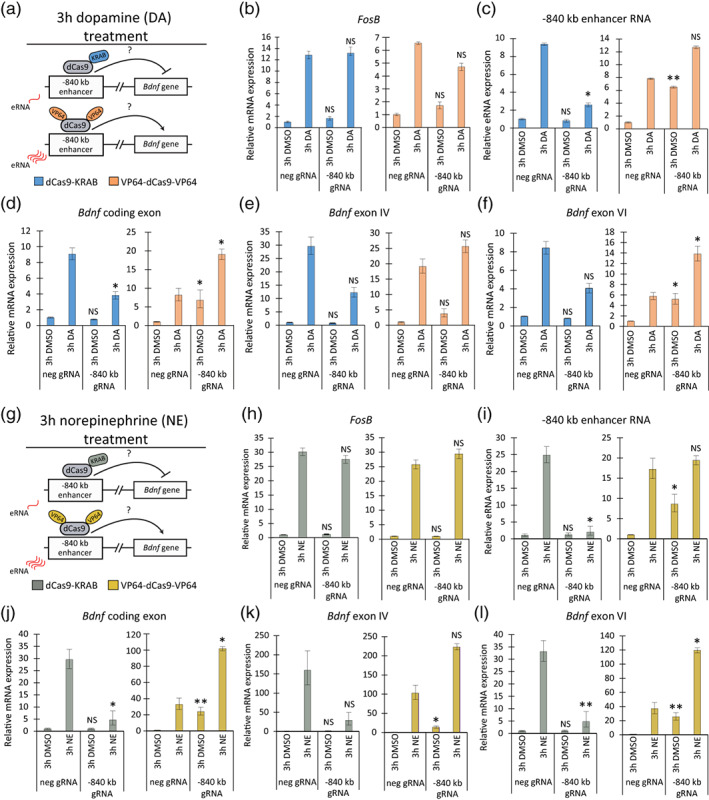
Dopamine‐ and norepinephrine‐induced *Bdnf* expression in rat cortical astrocytes is regulated by the −840 kb enhancer. (a, g) At 7 DIV, cultured rat cortical astrocytes were transduced with lentiviruses expressing dCas9‐KRAB or VP64‐dCas9‐VP64 and 5 gRNAs specific for the −840 kb enhancer region (−840 kb gRNA) or negative control gRNA (neg gRNA) as shown in the schematic. (b–f, h–l) At 15 DIV, astrocytes were treated for 3 h with 0.15% DMSO as a vehicle control or 150 μM dopamine (DA, b–f) or 25 μM norepinephrine (NE, h–l). The expression of *FosB* (b, h), −840 kb enhancer RNA (c, i), *Bdnf* coding exon (d, j) and *Bdnf* exon IV‐ (e, k) and exon VI‐containing (f, l) transcripts were measured using RT‐qPCR. The expression level of respective transcript in cells transduced with dCas9‐KRAB or VP64‐dCas9‐VP64 and negative gRNA‐encoding lentiviruses and treated with DMSO was set as 1. Error bars indicate SEM (*n* = 4 [b–f, *n* = 3 [h–l] independent experiments). Statistical significance was calculated with two‐tailed paired *t*‐test relative to the levels of respective transcript in cells infected with lentiviruses encoding negative gRNA and either dCas9‐KRAB or VP64‐dCas9‐VP64 upon respective treatment. NS, not significant, *p < .05; **p < .01; ***p < .001, *p*‐values were corrected for multiple comparisons with Holm‐Šidak method.

Next, we investigated the functionality of the −840 kb enhancer region to potentiate *Bdnf* expression in the endogenous context. We hypothesized that if the region under investigation enhances the transcription of *Bdnf* gene, modulating the activity of the enhancer should affect the expression of endogenous *Bdnf*. For this purpose, we used lentivirus‐encoded CRISPR interference and activator (CRISPRi/a) systems where a catalytically inactive Cas9 protein (dCas9) is fused with Krüppel associated box (KRAB) domain (dCas9‐KRAB) or 8 copies of VP16 domain (VP64‐dCas9‐VP64), respectively. For the enhancer‐specific repression or activation we used lentiviruses expressing five different gRNAs targeting the −840 kb region. As a control, we used lentiviruses expressing a gRNA that does not target any sequence in the rat genome. Cortical astrocytes were co‐transduced at 7 DIV with lentiviruses expressing dCas9‐KRAB or VP64‐dCas9‐VP64 and enhancer‐specific or negative control gRNAs (Figure 3a,g). At 15 DIV, rat cortical astrocytes were treated for 3 h with dopamine or DMSO as a vehicle control (Figure 3a–f). We also investigated the effect of −840 kb enhancer after norepinephrine treatment (Figure 3g–l), another catecholamine known to regulate *Bdnf* expression in astrocytes (Koppel et al., 2018). The −840 kb region eRNAs, mRNA levels of total *Bdnf* and different *Bdnf* transcripts were measured using RT‐qPCR.

To test the specificity of our system, we first measured the expression of *FosB*, an unrelated stimulus‐dependent gene. Neither repression nor activation of −840 kb enhancer region affected the expression of *FosB* (Figure 3b,h). The expression levels of −840 kb eRNAs were notably induced in cortical astrocytes—up to ~9‐fold upon dopamine (Figure 3c) and ~25‐fold after norepinephrine treatment (Figure 3i). Targeting dCas9‐KRAB complex to the −840 kb region remarkably decreased the levels of −840 kb eRNAs ~4‐ and ~12‐fold after dopamine and norepinephrine treatments, respectively, but did not affect the basal eRNA levels (Figure 3c,i). Activation of the −840 kb enhancer by VP64‐dCas9‐VP64 increased the −840 kb basal eRNA levels, but not stimulus‐dependent levels, up to ~9‐fold (Figure 3c,i). As the basal expression levels of the −840 kb eRNAs are very low, our data suggests that the −840 kb enhancer is not active in unstimulated rat astrocytes, but is activated upon dopamine and norepinephrine treatments. However, nascent bidirectional transcription from −840 kb region is evident in previously conducted GRO‐seq data obtained from human astrocytes (Figure S4) (Bouvy‐Liivrand et al., 2017), indicating that this region is transcriptionally active in unstimulated human glial cells. Further mechanistic experiments in human astrocytes would be necessary to unequivocally show that the −840 kb enhancer is active at basal levels and determine whether its activity differs in a species‐specific manner.

Repression of the −840 kb region with dCas9‐KRAB had no effect on the basal *Bdnf* mRNA expression level (Figure 3d–f,j–l), confirming the inactive/poised state of the enhancer at basal levels in rat cortical astrocytes. In contrast, dopamine‐ and norepinephrine‐induced levels of *Bdnf* exon IV‐ (Figure 3e,k) and VI‐containing transcripts (Figure 3f,l) were downregulated ~2‐ and ~6–7‐fold, respectively, after targeting dCas9‐KRAB to −840 kb enhancer region. Downregulation of different *Bdnf* transcripts was reflected in total *Bdnf* mRNA expression levels, which decreased ~2‐fold in dopamine (Figure 3d) and ~6‐fold in norepinephrine‐treated cells (Figure 3j). Activation of the −840 kb region increased the basal levels of *Bdnf* ~ 4–25‐fold and norepinephrine and dopamine‐dependent expression levels ~2–5‐fold (Figure 3d–f,j–l). Collectively, these results confirm the −840 kb region as a functional *Bdnf* gene enhancer in the endogenous context regulating catecholamine‐dependent but not basal expression of *Bdnf* in cortical astrocytes.

### 3.5 Deletion of the −840 kb enhancer decreases the catecholamine‐dependent expression of *Bdnf* in mouse embryonic stem cell‐derived astrocytes

**FIGURE 4:**
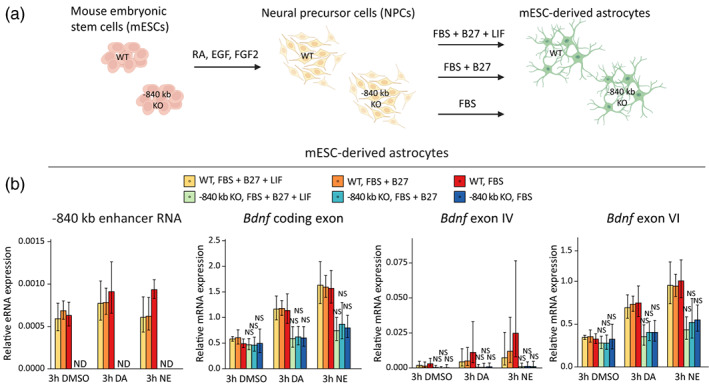
Deletion of the −840 kb enhancer region decreases the expression of *Bdnf* in mouse embryonic stem cell‐derived astrocytes. (a) CRISPR/Cas9 system was used to delete the ~500 bp conserved region of the −840 kb enhancer. Mouse embryonic stem cells with an intact −840 kb region (WT, *n* = 4 clones) or with a homozygous deletion of the −840 kb enhancer conserved core region (−840 kb KO, *n* = 3 clones) were differentiated to NPCs and then astrocytes using FBS alone or along with B27 (containing retinoic acid) or together with B27 and LIF as shown in the legend (illustration on panel a was created with BioRender.com). On the 21st day the induced astrocytes were treated for 3 h with 150 μM dopamine (DA) or 25 μM norepinephrine (NE) or with DMSO as a control. (b) The expression levels of −840 kb eRNAs, total *Bdnf* (*Bdnf* coding exon) or different *Bdnf* transcripts were measured using RT‐qPCR. The average mRNA expression level relative to the housekeeper *Cnot4* expression level is shown with error bars indicating SEM. Statistical significance was calculated with two‐tailed unpaired unequal variance *t*‐test relative to the expression level of the respective transcript in wildtype cells at the respective treatment. NS, not significant, #*p* < .1; *p < .05; **p < .01; ***p < .001, *p*‐values were corrected for multiple comparisons with Holm‐Šidak method. ND, not detected.

To confirm the endogenous function of the −840 kb enhancer, we deleted the enhancer region in mouse embryonic stem cells (mESCs). Based on genotyping results, wild‐type mESCs and clones with homozygous −840 kb enhancer deletion (Figure S5) were differentiated into neuronal precursor cells (NPCs) (Figure S6A,B) and astrocytes (Figure S7, Figure 4a). Because differentiation of astrocytes from mouse stem cells lacks a standardized protocol, we tried three different approaches that were selected based on previously published research, and we aimed to obtain astrocytes that show induction of *Bdnf* expression in response to dopamine and norepinephrine treatments. The differentiation strategies used either serum, serum with B27, or serum with B27 and LIF. These conditions led to the maturation of induced astrocytes in time (Figure S7A) and resulted in varied astrocyte morphology and marker gene expression (Figure S7B,C), possibly representing different subtypes and/or maturation stages of astrocytes. The cells were differentiated for 21 days to obtain astrocytes responding to dopamine and norepinephrine treatment. On the 21st day of differentiation the induced astrocytes were treated for 3 h with dopamine or norepinephrine and the expression levels of different *Bdnf* transcripts and −840 kb eRNA levels were measured using RT‐qPCR.

In NPCs, deletion of the −840 kb region abolished the expression of the eRNAs and slightly decreased the levels of total *Bdnf* and *Bdnf* exon VI‐containing transcripts (Figure S6C). In induced astrocytes, the deletion abolished the expression of −840 kb eRNAs and decreased the induction of total *Bdnf* ~ 2‐fold, *Bdnf* exon IV transcripts up to ~6‐fold and *Bdnf* exon VI‐containing transcripts ~2‐fold in both dopamine and norepinephrine treated astrocytes independent of the differentiation protocol (Figure 4b). However, the effects of *Bdnf* mRNA decreases were not statistically significant due to high biological variability between cell clones (Figure 4b). Similar to the endogenous repression of the enhancer in rat cultured astrocytes (Figure 3), deleting the −840 kb enhancer region did not decrease *Bdnf* expression in unstimulated mESC‐derived astrocytes, further corroborating that the −840 kb enhancer only participates in the regulation of *Bdnf* in stimulated cells. Collectively, homozygous deletion of the −840 kb core enhancer region decreased the catecholamine‐dependent expression of *Bdnf* in mESC‐derived astrocytes, providing further evidence for the functional significance of this element in the regulation of *Bdnf* expression.

### 3.6 CREB and AP1 family transcription factors regulate the −840 kb enhancer and *Bdnf* in rat cultured cortical astrocytes

**FIGURE 5:**
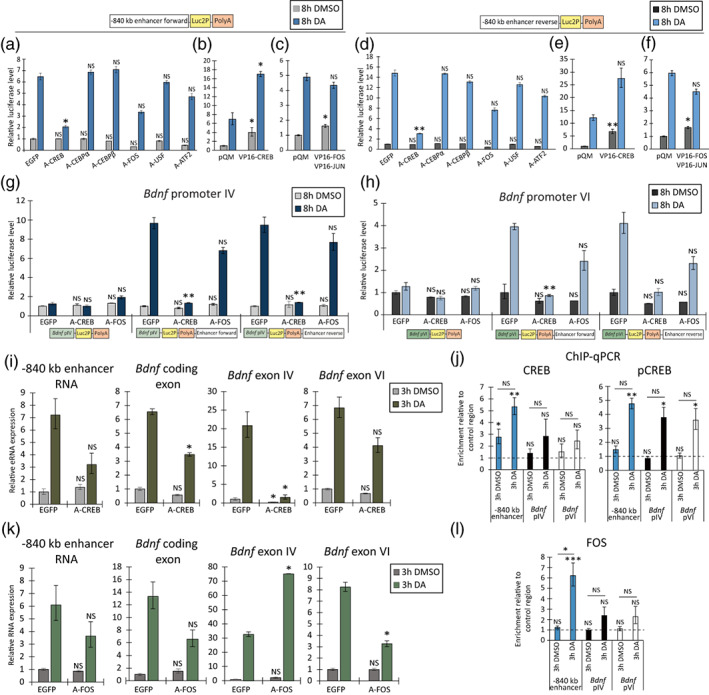
CREB and AP1 family transcription factors regulate the expression of *Bdnf* and −840 kb eRNA in rat cultured cortical astrocytes. (a–h) At 13 DIV, rat cortical astrocytes were cotransfected with different dominant‐negative transcription factors (termed as A in the name), constitutively active form of CREB (VP16‐CREB), FOS (VP16‐FOS), JUN (VP16‐JUN) or EGFP and luciferase reporter vectors as indicated under the graphs: −840 kb enhancer region in forward (a–c) or reverse (d–f) orientation before luciferase coding sequence or rat BDNF promoter IV (g) or VI (h) before luciferase coding sequence and −840 kb enhancer region in forward and reverse orientation after the polyadenylation signal (PolyA). Transfected astrocytes were treated for 8 h with 0.15% DMSO as control or with 150 μM dopamine (DA). The results are shown relative to the luciferase activity measured from DMSO‐treated astrocytes that were cotransfected with EGFP and respective reporter vector as shown below each panel. (i, k) At 7 DIV, rat cortical astrocytes were transduced with lentiviruses expressing either EGFP, dominant‐negative for the CREB family (A‐CREB, i), or dominant‐negative for the AP1 family (A‐FOS, k). At 15 DIV, astrocytes were treated with 0.15% DMSO as a control or 150 μM dopamine (DA) for 3 h. The RNA expression of −840 kb enhancer RNAs, total *Bdnf* (*Bdnf* coding exon), *Bdnf* exon IV‐ and exon VI‐containing transcripts was measured using RT‐qPCR. The expression level of the respective transcript in cells treated with DMSO and transduced with EGFP‐encoding lentiviruses was set as 1 (i, k). Error bars indicate SEM (*n* = 3 independent experiments). (j, l) Endogenous binding of CREB, pCREB and FOS was analyzed with ChIP‐qPCR. At 15 DIV, astrocytes were treated for 3 h with 0.15% DMSO as a control or 150 μM DA. A region ~5 kb downstream of the −840 kb enhancer was used as a negative control region. Data is depicted as enrichment relative to the control region (dashed line equals to 1). Error bars indicate SEM (*n* = 4 independent experiments). (a–h) Statistical significance was calculated using two‐tailed paired *t*‐test relative to the luciferase signal measured from respectively treated cells cotransfected with the respective reporter vector and EGFP plasmid (a–h), relative to the mRNA levels of respective transcript in respectively treated cells infected with lentiviruses encoding EGFP (i, k), or relative to the enrichment of DNA at the negative control region at respective treatment or the respective region in DMSO‐treated astrocytes (j, l). NS, not significant, *p < .05; **p < .01; ***p < .001; *p*‐values were corrected for multiple comparisons with Holm‐Šidak method.

To understand the molecular mechanism of the −840 kb enhancer in cortical astrocytes, we first used luciferase reporter assays to screen transcription factors (TFs) important for the transcriptional activity of the enhancer region. We hypothesized that if we inhibit the binding of TFs that are important for the activity of −840 kb region, then transcription from the enhancer region will decrease. To study this, rat cortical astrocytes were cotransfected with reporter vectors containing the −840 kb enhancer region in either forward (Figure 5a) or reverse orientation (Figure 5d) in front of the luciferase coding sequence together with a panel of dominant‐negative TFs, including A‐CREB, A‐CEBPα, A‐CEBPβ, A‐FOS, A‐USF, and A‐ATF2. Of note, all the selected and screened TFs have predicted binding elements in −840 kb region based on JASPAR computational prediction (Castro‐Mondragon et al., 2022). As a control, we cotransfected the EGFP‐encoding vector together with the −840 kb enhancer reporter construct. Transfected astrocytes were treated for 8 h with dopamine or DMSO as a vehicle control.

Basal transcriptional activity of the −840 kb region was decreased ~2–3‐fold in cells overexpressing A‐FOS, a dominant‐negative protein for AP1 family (Figure 5a,d). After dopamine treatment A‐FOS inhibited the activity of the −840 kb enhancer region ~2‐fold (Figure 5a,d). In addition to AP1 family, the dominant‐negative form of the CREB family, A‐CREB, almost abolished the induction of −840 kb transcription in cortical astrocytes upon dopamine treatment (Figure 5a,d). Other tested dominant negative proteins did not have a notable effect on the −840 kb region enhancer activity (Figure 5a,d). In addition, we overexpressed CREB or FOS and JUN fused to the VP16 activator domain together with −840 kb enhancer region to elucidate whether the CREB and AP1 family TFs can increase the transcription from the enhancer. The overexpression of constitutively active form of CREB (VP16‐CREB) robustly increased the transcriptional activity of the −840 kb enhancer both in DMSO‐ and dopamine‐treated cortical astrocytes (Figure 5b,e), while overexpression of VP16‐FOS and VP16‐JUN enhanced the transcriptional activity by ~2‐fold only in DMSO‐treated cortical astrocytes (Figure 5c,f), possibly due to saturation of −840 kb enhancer by endogenous AP1 family members after dopamine treatment.

Next, we studied whether the two dominant‐negative TFs (A‐CREB and A‐FOS) regulate the −840 kb enhancer region‐potentiated activation of *Bdnf* promoter IV and VI. For that, cells were cotransfected with A‐CREB and A‐FOS (Figure 5g,h) together with reporter constructs where *Bdnf* promoter IV (Figure 5g) or VI (Figure 5h) was in front of the luciferase coding sequence and the −840 kb enhancer region in forward or reverse orientation after the polyadenylation signal (PolyA). Overexpression of A‐CREB markedly decreased the −840 kb region‐enhanced activity of *Bdnf* promoter IV (Figure 5g) and VI (Figure 5h) in dopamine‐treated cortical astrocytes, whereas A‐FOS slightly decreased (~1.3–1.8‐fold) the activity of *Bdnf* promoter IV and VI (Figure 5g,h). These results indicate that CREB and AP1 family members mediate the activation of *Bdnf* promoters by the −840 kb enhancer region in a heterologous context in cortical astrocytes.

To investigate whether the TFs controlling the activity of the −840 kb region in the reporter assay also participate in the regulation of endogenous *Bdnf* gene expression, rat cortical astrocytes were transduced at 7 DIV with lentiviruses encoding either EGFP, A‐CREB (Figure 5i) or A‐FOS (Figure 5k). The overexpression of dominant‐negative CREB did not affect the basal expression levels of −840 kb eRNAs, but decreased the basal expression levels of exon IV‐containing *Bdnf* levels ~3‐fold (Figure 5i). Conversely, A‐CREB inhibited the expression of −840 kb eRNAs, total *Bdnf* and exon IV‐ and VI‐containing transcripts ~2–10‐fold in dopamine‐treated cells (Figure 5i). Using ChIP‐qPCR, we confirmed direct binding of CREB to −840 kb enhancer region in DMSO‐ and DA‐treated astrocytes (~3–5‐fold higher enrichment relative to a control region) and binding of the active form of CREB, phospho‐CREB (pCREB), which increased ~3‐fold following a dopamine treatment (Figure 5j). Overexpression of A‐FOS decreased the expression of −840 kb region eRNAs ~1.7‐fold in dopamine‐treated cells (Figure 5k). While overexpression of A‐FOS did not affect the basal levels of *Bdnf*, A‐FOS decreased total *Bdnf* and exon VI‐containing *Bdnf* mRNA (Figure 5k) levels ~2‐fold in dopamine‐treated astrocytes. Interestingly, *Bdnf* exon IV mRNA levels were upregulated ~2.3‐fold in dopamine‐treated A‐FOS overexpressing cells (Figure 5k). We also detected endogenous binding of FOS‐family TFs to the −840 kb enhancer region with ChIP‐qPCR (Figure 5l), where FOS binding increased ~5‐fold in dopamine‐treated cells. Although CREB and AP1 family TFs also showed some interaction with *Bdnf* promoters IV and VI, our results collectively point to the importance of the recruitment of these TFs to the −840 kb enhancer region for stimulating *Bdnf* gene expression in cortical astrocytes.

### 3.7 CREB and AP1 cis‐elements within the −840 kb enhancer are required for the enhancer activity

**FIGURE 6:**
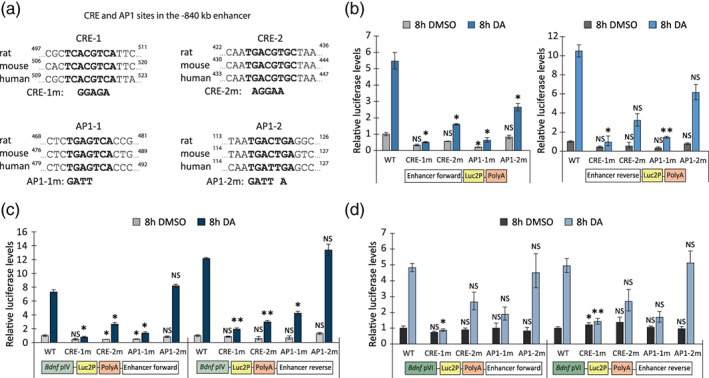
Mutations in the CRE and AP1 sites in the −840 kb region decrease the activity of the enhancer and *Bdnf* promoters in cultured rat cortical astrocytes. (a) CRE and AP1 sites in the rat, mouse and human −840 kb enhancer. Nucleotides shown in bold indicate the CREB or AP1 family transcription factor core binding sites, numbers indicate the sequence location counted from the 5′ of the cloned full‐length −840 kb enhancer region. Mutated nucleotides are shown below the wild‐type binding sites. (b–d) Rat cortical astrocytes were transfected at 13 DIV with luciferase reporter vectors indicated on the graphs: wild‐type (WT) or mutated rat −840 kb enhancer region in either forward or reverse orientation (b) or luciferase reporter constructs containing rat *Bdnf* promoter IV or VI upstream of the Luc2P coding sequence and the WT or mutated −840 kb region in either forward or reverse orientation downstream of the PolyA (c, d). Luciferase vector with WT enhancer region was used as a control. At 15 DIV, transfected astrocytes were treated for 8 h with 0.15% DMSO as a control or 150 μM dopamine (DA). The results are depicted relative to the luciferase signal measured from DMSO‐treated astrocytes transfected with WT enhancer construct (b–d). Error bars indicate SEM (*n* = 3 independent experiments). Statistical significance was calculated with two‐tailed paired *t*‐test relative to the luciferase level measured from DMSO‐treated cells transfected with luciferase vector with WT enhancer (b), or relative to the luciferase level measured from cells transfected with respective promoter‐containing vector with WT enhancer (c, d). NS, not significant, *p < .05; **p < .01; ***p < .001, *p*‐values were corrected for multiple comparisons with Holm‐Šidak method.

Finally, we examined the role of CREB and AP1 transcription factor binding sites in the transcriptional regulation of −840 kb enhancer. First, we used JASPAR cis‐element prediction of CREB and AP1 binding sites and found two CRE (cAMP responsive element, CRE‐1 and CRE‐2) and two AP1 sites (AP1‐1 and AP1‐2) in rat −840 kb enhancer (Figure 6a). Comparison of the sequences among rat, mouse and human revealed high evolutionary conservation of the identified binding sites, with the exception of AP1‐2, which differs by a single nucleotide in human compared to rat and mouse (Figure 6a). Next, we mutated the CREB and AP1 binding sites in the rat −840 kb enhancer reporter constructs and used luciferase reporter assay to study the importance of these sites in the bidirectional transcription of the enhancer.

In DMSO‐treated astrocytes, mutation only in the AP1‐1 site showed ~3–4‐fold downregulation of the −840 kb enhancer activity. The strongest stimulus‐dependent effect on the −840 kb enhancer activity was seen when mutating the CRE‐1 and AP1‐1 sites (Figure 6b)—the activity decreased ~11‐fold and ~7–9‐fold upon dopamine treatment, respectively. Mutations in CRE‐2 and AP1‐2 site decreased the stimulus‐dependent transcriptional activity ~2–3‐fold (Figure 6b).

Next, we analyzed whether the identified cis‐elements in the −840 kb enhancer affect the transcription from *Bdnf* promoters IV (Figure 6c) and VI (Figure 6d). Out of four tested cis‐elements, mutations in the CRE‐1, CRE‐2 and AP1‐1, but not in AP1‐2, decreased the dopamine‐dependent transcription from *Bdnf* promoter IV and VI. The strongest effect was seen with the mutations in the CRE‐1 site which decreased the dopamine‐dependent induction of *Bdnf* promoter IV ~6–9‐fold (Figure 6c) and *Bdnf* promoter VI ~4–5‐fold (Figure 6d). Taken together, these results show that all four identified transcription factor binding sites are responsible for the stimulus‐dependent activity of the rat −840 kb enhancer region, and specifically CRE‐1, CRE‐2 and AP1‐1 sites are necessary to increase the *Bdnf* promoter IV and VI activity after dopamine treatment in cortical astrocytes.

## 4 DISCUSSION

The multi‐promoter composition of *Bdnf* gene allows generating alternative *Bdnf* transcripts, which result in identical BDNF protein. This kind of gene regulation ensures complex spatiotemporal expression of BDNF (Esvald et al., 2023; West et al., 2014). For instance, the first cluster of *Bdnf* transcripts is expressed in neurons, but not in non‐neuronal cells, and, in contrast, the second cluster of *Bdnf* transcripts is expressed ubiquitously in both neuronal and non‐neuronal tissues (Aid et al., 2007; Esvald et al., 2023; Pruunsild et al., 2007; Timmusk et al., 1993). Likely, this spatiotemporal regulation is maintained by both cell type‐specific activity of proximal promoters (Palm et al., 1999; Zuccato et al., 2003) and recruitment of specific enhancer regions (Brookes et al., 2023; Flavell et al., 2008; Lyons et al., 2012; Tuvikene et al., 2021). In this study we aimed to identify distal regulatory regions that participate in the catecholamine‐dependent expression of *Bdnf* in rodent astrocytes. Of the four candidate enhancer regions that we initially investigated, only one region mapping 840 kb upstream of *Bdnf* gene showed properties of a stimulus‐dependent enhancer in astrocytes, and interaction with *Bdnf* promoter VI. It is possible that the rest of the bioinformatically predicted enhancer regions (−450, −40 and +37 kb) would function upon different stimuli or in another cell type than studied here, highlighting the uniqueness of each enhancer.

The astrocytic BDNF seems to have similar roles as neuron‐derived BDNF, for example, it modulates neuronal morphology, plasticity, and survival (De Pins et al., 2019; Giralt et al., 2010, 2011; Vignoli et al., 2016). However, in general the kinetics of exocytosis in astrocytes is slower compared to that in neurons (Kreft et al., 2004; Verkhratsky et al., 2016). Furthermore, due to different dynamics of BDNF secretion from neurons and astrocytes, the neuron‐ and astrocyte‐derived BDNF distinctively modulates the hippocampal long‐term potentiaton and learning (Liu et al., 2022). The physiological function of dopamine‐ and norepinephrine‐induced BDNF production in astrocytes remains to be investigated. In addition to neuron‐derived BDNF, it is plausible that astrocytes within the dopaminergic and noradrenergic tripartite synapses express BDNF to augment BDNF signaling, thereby contributing to several neuronal functions. What is the exact role of catecholamine signaling‐derived astrocytic BDNF in the cerebral cortex and whether the −840 kb enhancer facilitates the expression of astrocytic BDNF similarly in different brain regions, are important questions to address in the future.

Koppel et al. (2018) showed for the first time that the catecholamine‐dependent induction of *Bdnf* in astrocytes is not conferred only by the *Bdnf* proximal promoter regions. By using *BDNF*‐bacterial artificial chromosome (BAC)‐based reporters encompassing the whole rat or human *BDNF* gene locus, they found that at least some distal regulatory regions that facilitate the catecholamine‐induced levels of *Bdnf* in cortical astrocytes are located within or near the *Bdnf* gene. Notably, the −840 kb region described in the present study is outside of the *BDNF*‐BAC constructs used by Koppel et al. (2018). Together, these results show the existence of at least two enhancers, one 840 kb upstream of *Bdnf* described in the present study, and another within or nearby of *Bdnf* gene locus described by Koppel et al. (2018), that are responsible for catecholamine‐induced regulation of *Bdnf*. It is also plausible that *Bdnf* gene regulation in astrocytes is even more complex, considering the numerous long‐range chromatin interactions detected by our CAPTURE‐3C‐sequencing experiment. Namely, we determined that *Bdnf* pVI interacts with −840 kb region upon dopamine treatment, but we also showed dopamine‐dependent interactions with −990 kb and −2.65 Mb regions. Moreover, we found interaction with −790 kb region only in DMSO‐treated cells and a stimulus‐independent interaction with a region located at −1.82 Mb. This indicates that the transcription of *Bdnf* gene is subject to specific combinatorial regulation by different enhancer regions, as it has been demonstrated for *c‐Fos* (Joo et al., 2015). However, we acknowledge that our CAPTURE‐3C‐sequencing might lack the resolution and statistical power to detect the possible short‐range interactions within the *Bdnf* locus. We have previously shown that *Bdnf* is regulated as two clusters of promoters (Aid et al., 2007; Esvald et al., 2023; Pruunsild et al., 2007; Timmusk et al., 1993), and that the intronic enhancer located downstream of exon III is responsible for neuron‐specific expression of the first cluster of *Bdnf* exons (Tuvikene et al., 2021). It is plausible that the enhancers of *Bdnf* loop together with the anchor point located nearby the first, second, or both of the clusters depending on the cell type and stimulus. Further work is needed to determine the role and/or interplay of the potential enhancer regions to pinpoint the exact mechanism of the catecholamine‐dependent *Bdnf* expression.

Although the transcriptional regulation of *Bdnf* in astrocytes is poorly investigated, catecholamines have been shown to induce *Bdnf* gene expression in astrocytes via CREB‐dependent signaling (Jurič et al., 2008; Koppel et al., 2018). Our work provides further mechanistic insight by showing that AP1 family transcription factors act as regulators of astrocytic *Bdnf* expression. Interestingly, Koppel et al. (2018) showed that the well‐studied CRE element in *Bdnf* promoter IV is not involved in catecholamine‐dependent *Bdnf* regulation in astrocytes, whereas this site is critical for stimulus‐dependent expression of BDNF in neurons (Esvald et al., 2020; Hong et al., 2008; Pruunsild et al., 2011; Shieh et al., 1998; Tao et al., 1998). Similarly, AP1 family members are known to regulate the expression of the neuronal first cluster of *Bdnf* transcripts (Tuvikene et al., 2016) that are not expressed in astrocytes. These results argue for substantial differences in *Bdnf* gene regulation between different cell types even when the same transcription factor families are involved, implying involvement of other regulatory regions. Here, we demonstrate that the CREB and AP1 family‐dependent regulation of *Bdnf* in astrocytes occurs via the −840 kb enhancer region. We show that mutations in any of the CRE sites and one AP1 site within the −840 kb enhancer decrease the activity of *Bdnf* promoters IV and VI. Collectively, our study is the first report showing an enhancer coordinating the transcription of *Bdnf* gene in non‐neuronal cells and demonstrating the fine‐tuning of gene expression based on cellular context and signaling.

In neurons, the AP1 family plays a crucial role in the regulation of activity‐dependent enhancers. The AP1 motifs are one of the most prevalent cis‐elements in enhancers, and the regulation of neuronal enhancers relies on the rapid induction and binding of AP1 family transcription factors (Malik et al., 2014). The AP1 family acts as an epigenetic regulator, modifying chromatin accessibility around their binding sites throughout the genome (Malik et al., 2014; Su et al., 2017). To achieve this, the AP1 factors recruit ATP‐dependent BRG1/BRM associated factor (BAF) chromatin remodeling complexes, which, in turn, facilitate nucleosome remodeling and generate an accessible chromatin state (Vierbuchen et al., 2017). In our study, we identified two AP1 cis‐elements within the −840 kb enhancer region, both of which are significant for enhancer activity. However, only one of these AP1 cis‐elements (AP1‐1), located in the center of the −840 kb enhancer, is involved in enhancing the activity of *Bdnf* promoters. Interestingly, the other AP1 cis‐element (AP1‐2) appears to be non‐functional for *Bdnf* induction in astrocytes, but may play a crucial role in regulating *Bdnf* activity in other cell types. Currently, little is known about the general mechanism of enhancers in astrocytes, but our results studying this stimulus‐specific enhancer in depth suggests a similar central role for AP1 factors in astrocytes as observed in neurons. A natural continuation of this line of work would be to investigate the role of AP1 family transcription factors in astrocyte‐specific enhancers using genome‐wide approaches.

Notably, the −840 kb enhancer is relevant in vivo. First, in 2006, a case‐report of an 8‐year‐old girl suffering from hyperphagia, severe obesity, impaired memory and hyperactivity was published. Furthermore, it was determined that the BDNF protein levels were decreased in the patient's serum despite the fact that *BDNF* gene itself was not mutated (Gray et al., 2006). Genetic analysis revealed that the patient had a chromosomal inversion with a breakpoint located within a 9 kb region at the −840 kb enhancer locus. Second, a mouse line with transgene insertion in a conserved region located approximately 840 kb upstream of the *Bdnf* gene (Sha et al., 2007) was reported to have a similar phenotype as *Bdnf* knockout mice (Lyons et al., 1999; Rauskolb et al., 2010) and patient described in Gray et al. (2006). Furthermore, the transgene insertion strikingly reduced both *Bdnf* mRNA and protein levels in the hypothalamus and in the hippocampus (Sha et al., 2007). However, neither of these studies unequivocally showed the exact enhancer region responsible for the decreased BDNF levels and the observed phenotype. Our current study provides one possible explanation for these findings by describing a functional enhancer in the −840 kb region regulating *Bdnf* transcription in astrocytes. Further dissecting the in vivo function and role of this enhancer in various cell types would improve the understanding of *Bdnf* gene regulation. Collectively, the phenotypic abnormalities in human and mice caused by alteration in the −840 kb region (Gray et al., 2006; Sha et al., 2007) should be considered as an example of enhanceropathy, a condition caused by a misregulation of an enhancer regulating a functionally important gene (Claringbould & Zaugg, 2021; Smith & Shilatifard, 2014).

In conclusion, our study provides the first description of an enhancer element regulating stimulus‐specific transcription of *Bdnf* in an important type of non‐neuronal cells. Whether the knowledge of the molecular mechanisms underlying BDNF production in astrocytes can be harnessed for therapeutic applications is an important question for the future.

## AUTHOR CONTRIBUTIONS

AA designed research, performed experiments, wrote the first draft, and edited the manuscript. E‐EE, IK and JT designed research, performed experiments, wrote and edited the manuscript. JT performed bioinformatical analysis. AP performed experiments and reviewed the manuscript. AZ and EVM provided funding, and edited the manuscript. TT and JT conceived the idea, supervised the study, TT provided funding, and edited the manuscript. All authors contributed to the article and approved the submitted version.

## CONFLICT OF INTEREST STATEMENT

Eli‐Eelika Esvald, Jürgen Tuvikene, and Tõnis Timmusk were employees of Protobios LLC. The authors declare no other competing financial interests.

## Supporting information


**Appendix S1** Supporting information.

**Figure S1** Supporting information.

## Data Availability

The data that support the findings of this study are available from the corresponding author upon reasonable request.
