# Supplementary material for: An 840 kb distant upstream enhancer is a crucial regulator of catecholamine‐dependent expression of the Bdnf gene in astrocytes
Source: Glia. 2023 Aug 25;72(1):90–110. doi: 10.1002/glia.24463 (PMC10952894; doi:10.1002/glia.24463)
Supplement: Supplementary file 2 — Figure S1 Supporting information. [file GLIA-72-90-s002.docx]

**Supporting information**

**
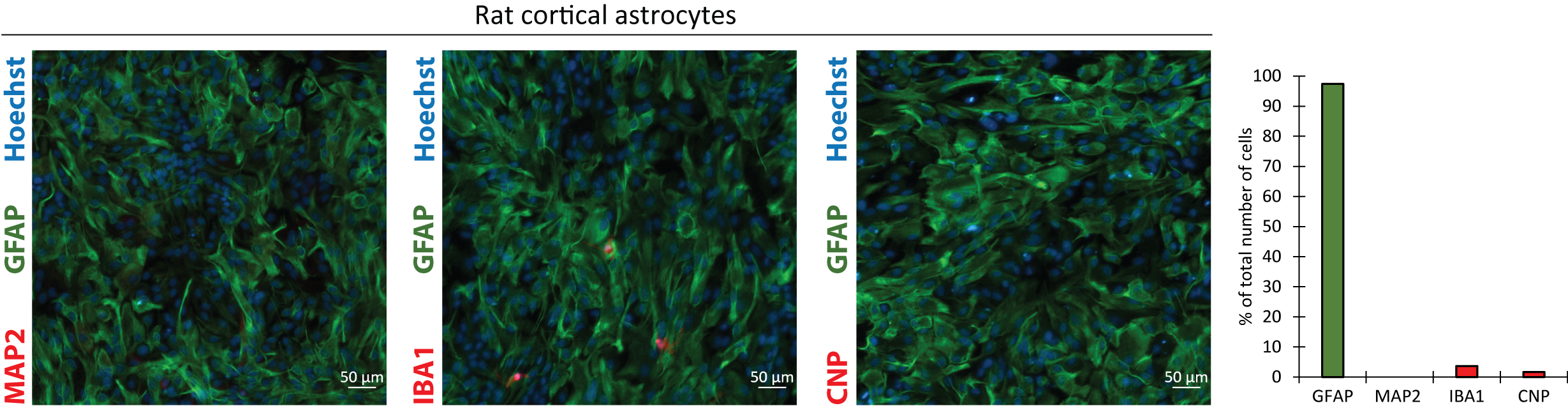
**

**Figure S1. The cultured rat cortical astrocytes are GFAP positive by immunocytochemical analysis.** At 15 DIV, rat cortical astrocytes were stained using anti-GFAP (astrocytes), anti-MAP2 (neurons), anti-IBA1 (microglia), anti-CNP (oligodendrocytes) antibody, and Hoechst 33342 to visualize nuclei. Total number of cells was counted using Hoechst 33342 staining in ImageJ program and immunocytochemically labelled cells were counted manually. >95% of the cells were GFAP positive.


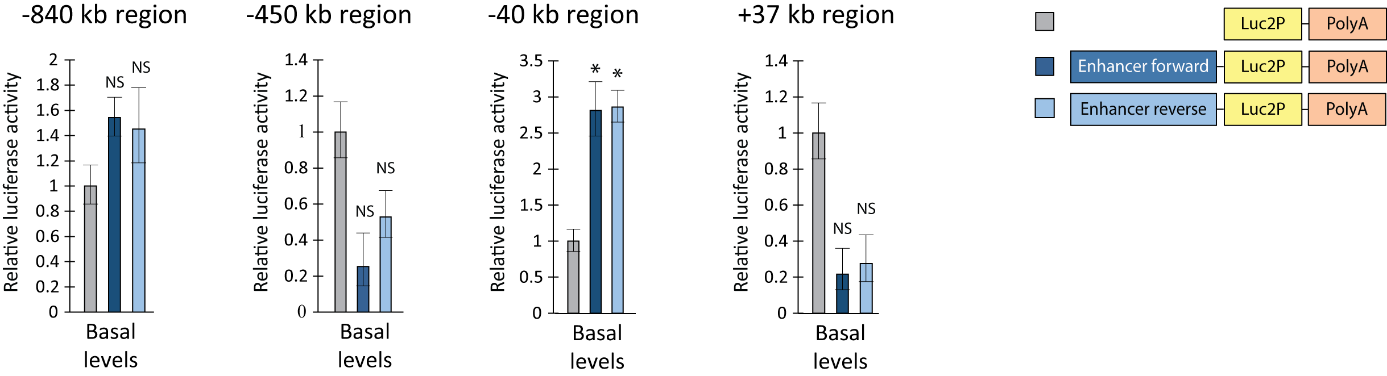


**Figure S2. The -40 kb enhancer region shows the strongest bidirectional transcription in DMSO-treated rat cortical astrocytes.** At 13 DIV, rat cortical astrocytes were transfected with luciferase reporter vectors indicated next to the graph: rat -840 kb, -450 kb, -40 kb or +37 kb putative enhancer region in either forward or reverse orientation in front of the luciferase coding sequence (Luc2P) followed by polyadenylation signal (PolyA). Luciferase vector without an enhancer region was used as a negative control. At 15 DIV, transfected astrocytes were treated for 8h with 0.15% DMSO. Results are shown relative to the luciferase activity measured from cells transfected with the luciferase vector without enhancer. Error bars indicate SEM (n=3 independent experiments). The data underlying this figure is the same as in Figure 1B. Statistical significance was calculated with two-tailed paired t-test relative to the luciferase level measured from cells transfected with luciferase vector containing no promoter region. NS: not significant, *p < 0.05, **p < 0.01, ***p < 0.001, p-values were corrected for multiple comparisons with Holm-Šidak method.


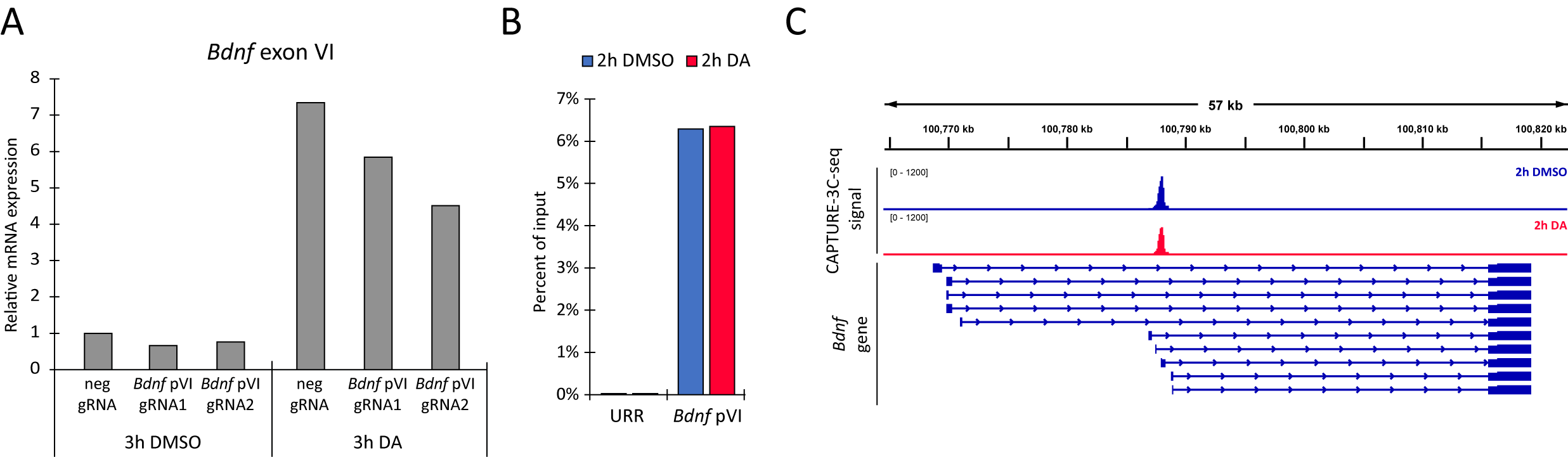


**Figure S3. The effect of targeting CRISPR-dCas9 system to *Bdnf* promoter VI on *Bdnf* expression levels and *Bdnf* promoter VI enrichment in CAPTURE-3C-sequencing experiment.** (**A**) Astrocytes were transduced at 7 DIV with different gRNAs, FB-dCas9 and BirA, and treated with DMSO or 150 µM dopamine (DA) for the indicated time at 19 DIV. The levels of *Bdnf* exon VI-containing transcripts were measured using RT-qPCR. (**B**) For CAPTURE-3C, the astrocytes were transduced with lentiviruses encoding gRNAs 1 and 2, BirA and FB-dCas9, and treated at 15 DIV with DMSO or DA for 2h. The enrichment of *Bdnf* promoter VI (pVI) and unrelated region (URR) after CAPTURE-3C protocol and before library preparation were measured with qPCR. Enrichment is shown relative to the respective DNA levels in input samples. (**C**) Graphical visualization of the CAPTURE-3C-sequencing reads mapping to the Bdnf locus show notable read enrichment at the *Bdnf* pVI anchor region.


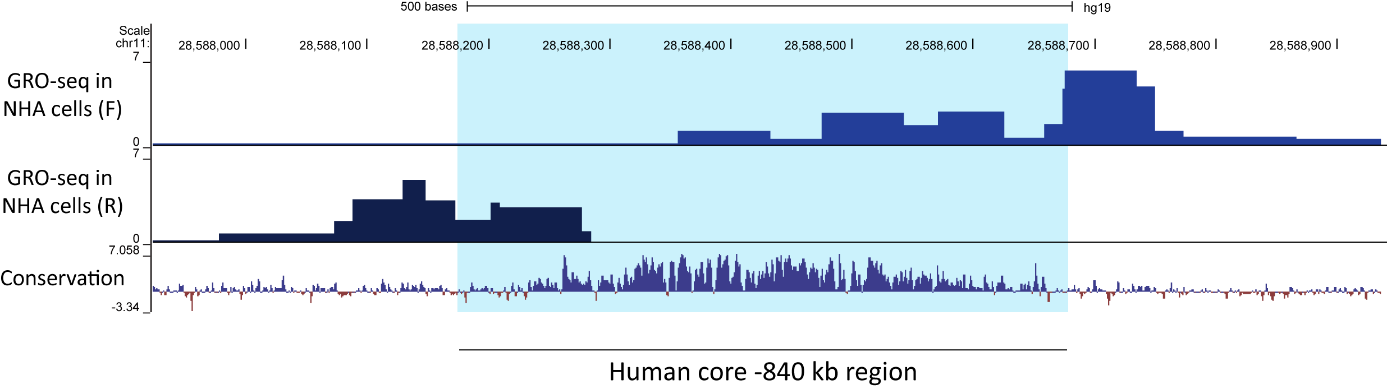


**Figure S4. The human -840 kb enhancer region shows bidirectional transcription in human astrocytes.** UCSC Genome Browser tool was used to depict the bidirectional transcription in the -840 kb enhancer region. The GRO-seq track shows nascent transcription by RNA polymerase II in normal human astrocyte (NHA) cells on both forward (F) and reverse (R) strand (Bouvy-Liivrand et al., 2017). The conservation track indicates the conservation in vertebrates.


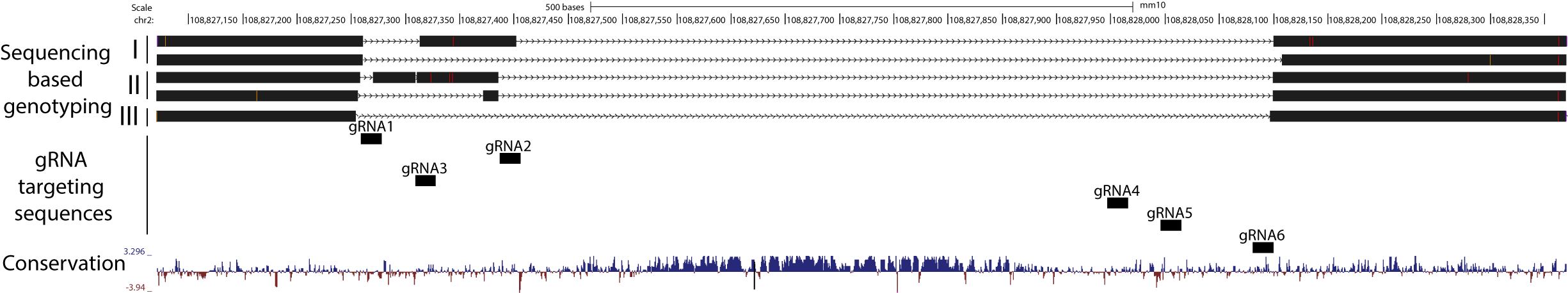
 **Figure S5. Sanger sequencing-based genotyping of the mouse embryonic stem cell clones where -840 kb enhancer region was deleted.** The sequences of PCR amplicons of three different mESC clones (denoted with roman numerals I, II and III) with -840 kb enhancer deletion are shown using UCSC Genome Browser. Clones I and II showed different -840 kb enhancer deletion between alleles; clone number III had an identical deletion in both alleles. Dotted line indicates deletion and black boxes show matching regions compared to the genomic DNA in the -840 kb enhancer locus. gRNA targeting sequences (gRNA1-6) that were used for deleting the -840 kb enhancer region are shown below. The conservation track indicates the conservation of the region in vertebrates.


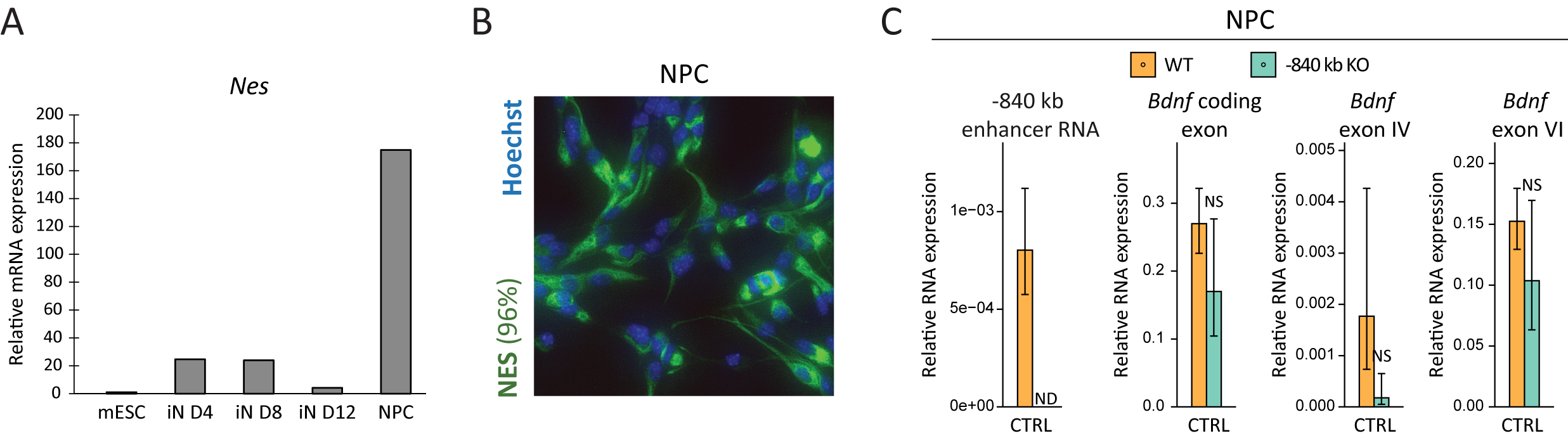


**Figure S6. Mouse embryonic stem-cell derived neuronal precursor cells show expected morphology and *Nes* expression, and slightly reduced levels of *Bdnf* mRNA in cell lines with deletion of the -840 kb enhancer region.** (**A**) *Nes* mRNA levels were measured with RT-qPCR from mouse embryonic stem cells (mESCs), induced neurons (iN) at different days after starting the differentiation (day 4 (D4), day 8 (D8), or day 12 (D12) (differentiated using *Ngn2* overexpression as described in Tuvikene et al., 2021), and in neuronal precursor cells (NPC-s). The *Nes* mRNA levels are shown as relative to the amount of RNA taken for cDNA synthesis and relative to *Nes* expression levels in mESCs. (**B**) The neuronal precursor cells (NPCs) were described with immunocytochemistry using anti-NES antibody. Based on the immunocytochemical analysis, 96% of the cells were positive for NESTIN. (**C**) The levels of enhancer RNAs from -840 kb region and *Bdnf* mRNA levels were measured using RT-qPCR. The graph indicates results of wildtype (n=4) and homozygous knockout (n=3) cell lines. The average mRNA expression level relative to *Cnot4* expression level is shown with error bars indicating SEM. Statistical significance was calculated with two-tailed unpaired unequal variance t-test relative to the expression level of the respective transcript in wildtype cells. NS: not significant, #< 0.1, *p < 0.05, **p < 0.01, ***p < 0.001, p-values were corrected for multiple comparisons with Holm–Šidak method. ND: not detected.

**
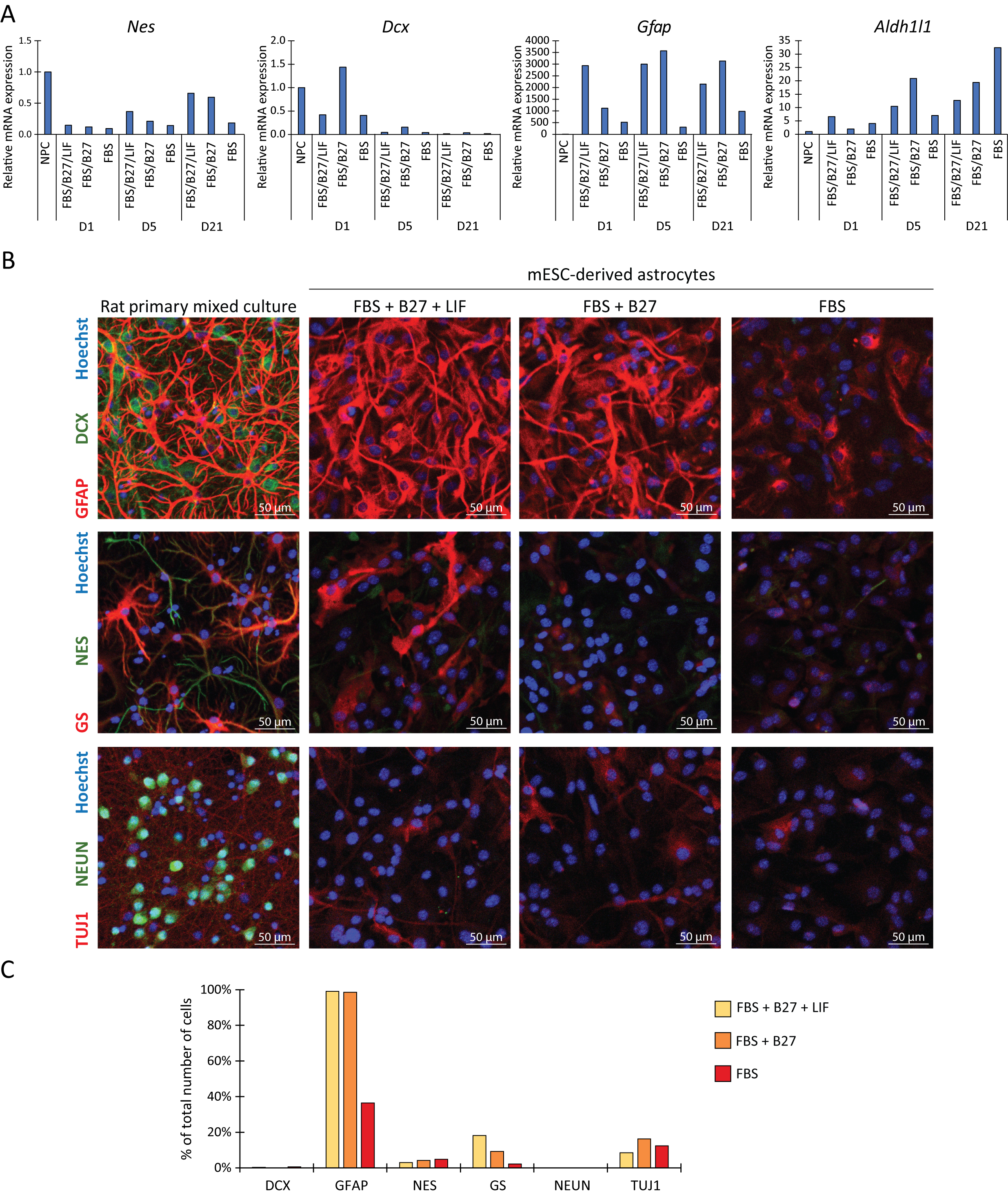
**

**Figure S7.** **Characterization of the mouse embryonic stem cell-derived astrocyte cultures obtained using different differentiation protocols.** The mESC-derived astrocytes were induced using different protocols. (**A**) Marker gene expression levels were analysed at different times with RT-qPCR. The relative marker gene mRNA levels in induced astrocytes after 1 day (D1), 5 days (D5), and 21 days (D21) of differentiation are shown relative to the respective gene expression levels in neuronal precursor cells (NPC-s). (**B**) The induced astrocytes were characterized using immunocytochemistry at 21^st^ day of differentiation in parallel with primary mixed culture to validate the functionality of the used antibodies. The cells were analysed using antibodies marking neuronal precursor cells and immature neuronal cells (anti-NES, anti-DCX), astrocytes (anti-GFAP, anti-GS), neurons (anti-NEUN, anti-β-III-Tubulin (TUJ1)), and Hoechst 33342 to visualize nuclei. The fluorescence signal was captured using the same microscope parameters for primary culture and induced cells. (**C**) Quantification of immunocytochemistry of induced astrocytes. Total number of cells was counted using Hoechst 33342 staining in ImageJ program and immunocytochemically labelled cells were counted manually.
